# Hey1- and p53-dependent TrkC proapoptotic activity controls neuroblastoma growth

**DOI:** 10.1371/journal.pbio.2002912

**Published:** 2018-05-11

**Authors:** Marie Ménard, Clélia Costechareyre, Gabriel Ichim, Jonathan Blachier, David Neves, Loraine Jarrosson-Wuilleme, Reinhard Depping, Jan Koster, Pierre Saintigny, Patrick Mehlen, Servane Tauszig-Delamasure

**Affiliations:** 1 Apoptosis, Cancer and Development Laboratory—Equipe labellisée ‘La Ligue’, LabEx DEVweCAN, Centre de Recherche en Cancérologie de Lyon, INSERM U1052-CNRS UMR5286, Université de Lyon, Centre Léon Bérard, Lyon, France; 2 Centre de Recherche en Cancérologie de Lyon, INSERM U1052-CNRS UMR5286, Université de Lyon, Centre Léon Bérard, Lyon, France; 3 Universität zu Lübeck, Institut für Physiologie, Zentrum für Medizinische Struktur und Zellbiologie, Lübeck, Germany; 4 Department of Oncogenomics, Academic Medical Center, Amsterdam, the Netherlands; 5 Department of translational Research and Innovation, Centre Léon Bérard, Lyon, France; St. Jude Childrens Research Hospital, United States of America

## Abstract

The neurotrophin-3 (NT-3) receptor tropomyosin receptor kinase C (TrkC/NTRK3) has been described as a dependence receptor and, as such, triggers apoptosis in the absence of its ligand NT-3. This proapoptotic activity has been proposed to confer a tumor suppressor activity to this classic tyrosine kinase receptor (RTK). By investigating interacting partners that might facilitate TrkC-induced cell death, we have identified the basic helix-loop-helix (bHLH) transcription factor Hey1 and importin-α3 (karyopherin alpha 4 [KPNA4]) as direct interactors of TrkC intracellular domain, and we show that Hey1 is required for TrkC-induced apoptosis. We propose here that the cleaved proapoptotic portion of TrkC intracellular domain (called TrkC killer-fragment [TrkC-KF]) is translocated to the nucleus by importins and interacts there with Hey1. We also demonstrate that Hey1 and TrkC-KF transcriptionally silence mouse double minute 2 homolog (MDM2), thus contributing to p53 stabilization. p53 transcriptionally regulates the expression of TrkC-KF cytoplasmic and mitochondrial interactors cofactor of breast cancer 1 (COBRA1) and B cell lymphoma 2–associated X (BAX), which will subsequently trigger the intrinsic pathway of apoptosis. Of interest, TrkC was proposed to constrain tumor progression in neuroblastoma (NB), and we demonstrate in an avian model that TrkC tumor suppressor activity requires Hey1 and p53.

## Author summary

Tropomyosin receptor kinase C (TrkC) is a transmembrane receptor at the cell surface and has been described to work paradoxically both as an oncogene and as a tumor suppressor. We partly solved this paradox in a previous study, demonstrating that TrkC is a double-facet receptor: Upon interaction with its ligand neurotrophin-3 (NT-3), TrkC has a tyrosine kinase activity and induces survival and proliferation of the cell; conversely, in the absence of the ligand, TrkC is cleaved and releases a "killer-fragment" that triggers apoptosis. In this study, we analyze the fate of this fragment and show that TrkC killer-fragment is translocated to the nucleus, where it stabilizes the apoptosis inducer p53. We further find that p53 activates the transcription of cytoplasmic molecular partners, which interact with TrkC killer-fragment and induce apoptosis. We also demonstrate that alteration of this mechanism favors tumor growth in neuroblastoma (NB), an avian tumor progression model for a pediatric cancer.

## Introduction

The neurotrophins nerve growth factor (NGF), brain-derived neurotrophic factor (BDNF), neurotrophin-3 (NT-3), NT-4/5, and their respective receptors neurotrophin receptor p75 (p75^NTR^) and tropomyosin receptor kinases (TrkA), B, and C have been notably studied for their critical role in neurodevelopment [1]. Yet as TrkA, B, and C are tyrosine kinase receptors (RTKs), their deregulated functions in cancer have been investigated [2]. The overall view is that their kinase activity confers them the ability to activate mitogen-activated protein kinase (MAPK) and phosphoinositide 3-kinase (PI3K)/AKT pathways known to promote cell survival, proliferation, and differentiation under physiological conditions and to contribute to tumor progression when constitutively activated in cancers [2]. The kinase domains of TrkA, B, and C are indeed involved in oncogenic translocations or mutated in cancers (for review [2]). In line with the pharmaceutical rush to design antitumoral treatments based on RTK inhibition, drugs targeting TrkA, B, and C have been under development [3]. Nevertheless, TrkC expression has been paradoxically associated with favorable outcome in pediatric neoplasia, namely neuroblastoma (NB) and medulloblastoma, and was more recently shown to act as a tumor suppressor in colon cancer ([4] and for review [5–8]). We and others have indeed proposed that TrkC has a dual functionality: (i) In presence of its ligand NT-3, TrkC behaves as a classical RTK, transducing positive signals; (ii) in absence of NT-3, TrkC does not stay inactive but rather triggers apoptosis [9, 10]. TrkC thus belongs to the functional family of "dependence receptors." These receptors play a crucial role in constraining the adequate number of cells in a tissue in which the ligand is expressed in a limited amount during neurodevelopment but also during tumorigenesis: Cells in excess that carry an unbound dependence receptor undergo apoptosis [11]. It was demonstrated in different types of tumors that (i) the silencing of the dependence receptor by epigenetic mechanisms or genetic alterations or (ii) the overexpression of the ligand confers to the tumor cells a survival selective advantage: The dependence receptor is then no longer able to trigger apoptosis. TrkC expression was indeed shown to be epigenetically silenced in colon tumors [4, 6]. Along the same line, we also demonstrated that a large proportion of high-grade NB tumors shows an autocrine production of NT-3 as a mechanism to constitutively block TrkC proapoptotic function. It was thus proposed that interfering with ligand–receptor (NT-3/TrkC) interaction, either by gene silencing or the use of a blocking antibody, is associated in different animal models with the inhibition of tumor growth and metastasis [12].

The mechanism for TrkC proapoptotic activity has been investigated in recent years [9, 10, 13]. Upon ligand withdrawal, TrkC appears to be cleaved by caspase-like proteases at 2 sites (D495 and D641) within its intracellular domain, and the released fragment (TrkC 496–641, called the "killer-fragment" [TrkC-KF]) is necessary and sufficient to promote apoptosis. We demonstrated recently that this fragment interacts with cofactor of breast cancer 1 (COBRA1), which shuttles TrkC-KF to the mitochondria [13]. Once at the mitochondria, TrkC-KF and COBRA1 activate B cell lymphoma 2–associated X (BAX) and induce mitochondrial outer membrane permeabilization (MOMP), the release of cytochrome *c*, and the subsequent apoptosome activation [13].

Here, we show that TrkC-KF is not only cytoplasmic as described previously but is also observed in the nucleus. TrkC-KF is translocated to the nucleus by importins. A 2-hybrid screen allowed us to identify that TrkC-KF then interacts with Hey1, a basic helix-loop-helix (bHLH) transcription factor originally described as an effector of the NOTCH pathway. Hey1 and TrkC-KF bind on mouse double minute 2 homolog (*MDM2*) promoter and negatively regulate *MDM2* transcription. This decrease of MDM2 expression favors p53 stabilization, which triggers the transcription of TrkC proapoptotic partners acting at the mitochondria. We finally show in an avian model of NB tumor progression that Hey1- or p53-silencing abrogates TrkC tumor suppressor activity.

## Results

### Importins mediate the nuclear translocation of TrkC proapoptotic fragment TrkC-KF

**Fig 1:**
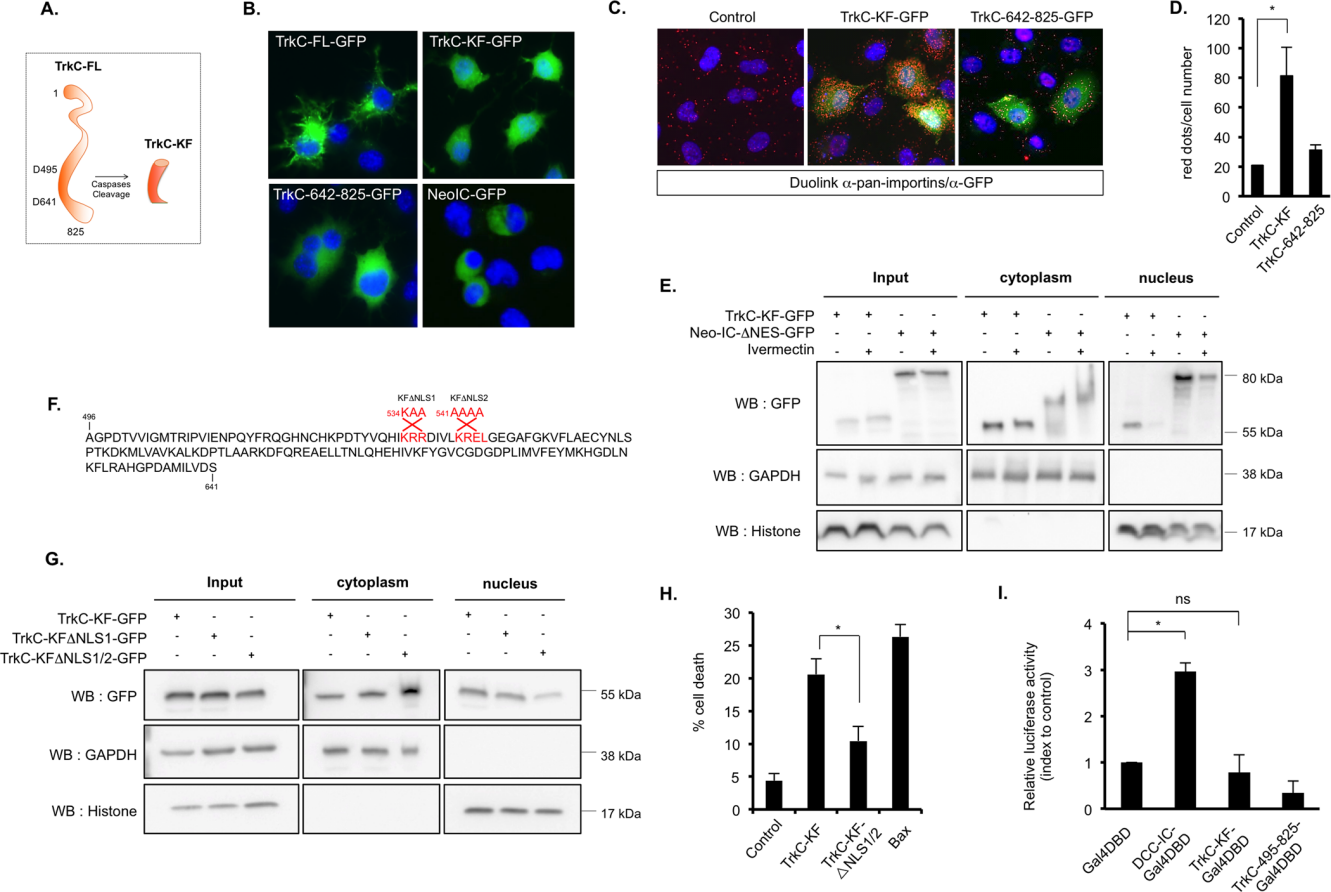
TrkC-KF is translocated to the nucleus by importins. (A) Schematic representation of TrkC-KF, generated after TrkC cleavage by caspase in D495 and D641, in absence of NT-3. A second fragment, TrkC 642–825, is also generated but has no apoptotic activity. (B) Expression of TrkC-FL, TrkC-KF, TrkC 642–825, and Neo-IC fused to GFP in N2A murine neuroblastoma cells. Nuclei are stained with DAPI. (C) Proximity ligation assay (DuoLink) using a pan-importins antibody and an anti-GFP antibody on untransfected SHEP cells or those transfected with TrkC-KF-GFP or TrkC-642-825-GFP: The protein–protein interactions are visualized by red fluorescent spots (Cy3). (D) Quantification of the proximity ligation assay presented in (C): Data represent mean ± SEM (at least 2 independent fields). *p < 0.05. *t* test compared with control (untransfected). (E) SHEP cells transfected with either TrkC-KF or Neo- IC-ΔNES and treated or not with 10 μM of pan-importin inhibitor Ivermectin were fractionated into cytoplasmic (Cytoplasm, marker: GAPDH) and nuclear (Nucleus, marker: histone) fractions. (F) Localization of the 2 putative NLSs in TrkC-KF sequence: Mutations of these NLSs used in experiment (G) are indicated in red. TrkC-KF-ΔNLS1/2 is mutated on both NLS1 and NLS2. (G) SHEP cells transfected with the NLS-mutated constructs of TrkC-KF were fractionated into cytoplasmic (Cytoplasm, marker: GAPDH) and nuclear (Nucleus, marker: histone) fractions. (H) Cell death quantification by trypan blue exclusion after transfection of SHEP cells with TrkC-KF, TrkC-KF-ΔNLS1/2, or Bax. Data represent mean ± SEM (*n* = 3). *p < 0.05. *t* test. (I) Quantification of luciferase activity in SHEP cells transfected with DCC-IC, TrkC-KF, or TrkC-492-825 constructs fused to the Gal4 DBD, and the gene coding for luciferase under the UAS-Gal4 control. Data represent mean ± SEM. *p < 0.05. *t* test compared to control (Gal4-DBD). Underlying data can be found in S1 Data. Bax, B cell lymphoma 2–associated X; DBD, DNA-binding domain; DCC-IC, deleted in colorectal cancer intracellular domain; GAPDH, glyceraldehyde 3-phosphate dehydrogenase; GFP, green fluorescent protein; N2A, Neuro2a; Neo-IC, intracellular fragment of Neogenin; Neo-IC-ΔNES, Neo-IC deleted for its nuclear export sequence; NES, nuclear export sequence; NLS, nuclear localization sequence; ns, nonsignificant; NT-3, neurotrophin-3; TrkC, tropomyosin receptor kinsase C; TrkC-FL, full-length TrkC; TrkC-KF, TrkC killer-fragment; UAS, upstream activating sequence; WB, western blot.

We have previously shown that in absence of its ligand NT-3, TrkC is cleaved by caspase at 2 sites (D495 and D641) within its intracellular domain, leading to the release of several intracellular fragments. This caspase-dependent cleavage can be detected both in vitro and in vivo and is required for apoptosis induction, since the mutation of the caspase sites inhibits apoptosis induced by TrkC [9, 12, 13]. TrkC cleavage by caspases leads to the generation of 3 fragments: TrkC 1–495, TrkC 496–641, and TrkC 642–825 (Fig 1A). In various cell lines, including the murine Neuro2a (N2A) and human SHEP NB cell lines enforced expression of the internal caspase-generated fragment TrkC 496–641 (named TrkC-KF) was associated with cell death induction, while TrkC 1–495 and TrkC 642–825 displayed no proapoptotic activity [9, 13]. In addition to its mitochondrial localization described earlier [13], the green fluorescent protein (GFP)–tagged TrkC-KF (TrkC-KF-GFP) was detected in the nucleus of N2A cells (Fig 1B). As a control, full-length TrkC (TrkC-FL-GFP), the C-terminal cleavage fragment (TrkC-642-825-GFP), and the intracellular fragment of an unrelated receptor—Neogenin (Neo-IC-GFP)—were mostly detected outside the nucleus of transfected cells (Fig 1B). We used GFP-fused fragments as none of the commercial antibodies or antibodies we generated were able to detect endogenous TrkC-KF. We verified that Flag-tagged TrkC-KF was also observed both in the cytoplasm and in the nucleus upon cellular fractionation (S1A Fig). In a yeast 2-hybrid screen using TrkC-KF as bait and a mouse embryonic cDNA library as prey, we identified importin-α3 (karyopherin alpha 4 [KPNA4]) (S1B Fig) [13]. Importins are cargo proteins shuttling cytoplasmic proteins into the nucleus [14, 15]. A proximity ligation assay (Duolink) using a pan-importin antibody and an anti-GFP antibody allowed us to detect a close interaction between TrkC-KF-GFP and endogenous importins (Fig 1C and 1D), suggesting that TrkC-KF is interacting with importins. We thus treated N2A cells with Ivermectin, a pan-importin inhibitor, and performed a fractionation experiment (Fig 1E and S1C Fig). As a control, we used a version of Neo-IC deleted for its nuclear export sequence (Neo-IC-ΔNES) but with an intact nuclear localization sequence (NLS), which is mostly localized in the nucleus [16]. We observed that the amount of TrkC-KF was greatly reduced in the nucleus upon treatment with Ivermectin, while the cytoplasmic pool was not significantly affected (Fig 1E and S1C Fig). As a positive control, Neo-IC nuclear translocation was also affected by Ivermectin treatment. TrkC-KF thus appears to be shuttled in the nucleus by importins. Importins need to first recognize NLSs in the proteins they are supposed to shuttle [14, 15]. Two putative NLSs could be mapped in TrkC-KF sequence, and we thus generated constructs bearing 1 (KFΔNLS1) or the 2 (KFΔNLS1/2) mutations of these putative sites (Fig 1F). While the mutation of NLS1 had no effect on TrkC-KF nuclear translocation, mutation of NLS1/2 greatly reduced the amount of TrkC-KF in the nuclear fraction of transfected SHEP cells (Fig 1G and S1D Fig). Furthermore, the mutation of NLS1/2 (TrkC-KFΔNLS1/2) is sufficient to partially but significantly inhibit TrkC proapoptotic activity (Fig 1H) without affecting its functionality. Indeed, TrkC-KFΔNLS1/2 is able to bind COBRA1, its cytoplasmic partner, as wild-type TrkC-KF does when overexpressed in cells (S1E Fig), suggesting that this mutant is still functional. TrkC-KF nuclear translocation seems therefore necessary for its proapoptotic activity in SHEP cells. In addition, no nuclear export sequence (NES) could be found in TrkC-KF, suggesting that once in the nucleus, TrkC-KF does not return in the cytoplasm. To monitor the role of TrkC-KF in the nucleus, we investigated whether it is able to transactivate gene transcription. To first assay this, TrkC-KF was fused to a Gal4 DNA-binding domain (DBD), and SHEP cells were forced to express TrkC-KF-Gal4DBD together with a construct encoding a luciferase reporter gene under the control of the upstream activating sequence (UAS)-GAL4 promoter. As shown in Fig 1I and S1F Fig, TrkC-KF-Gal4DBD is unable to transactivate the UAS-GAL4, unlike deleted in colorectal cancer intracellular domain (DCC-IC), as shown previously [17]. This result suggests that TrkC-KF has no intrinsic transcriptional activity per se.

### The transcription factor Hey1 interacts with TrkC-KF in the nucleus and allows its binding to chromatin

**Fig 2:**
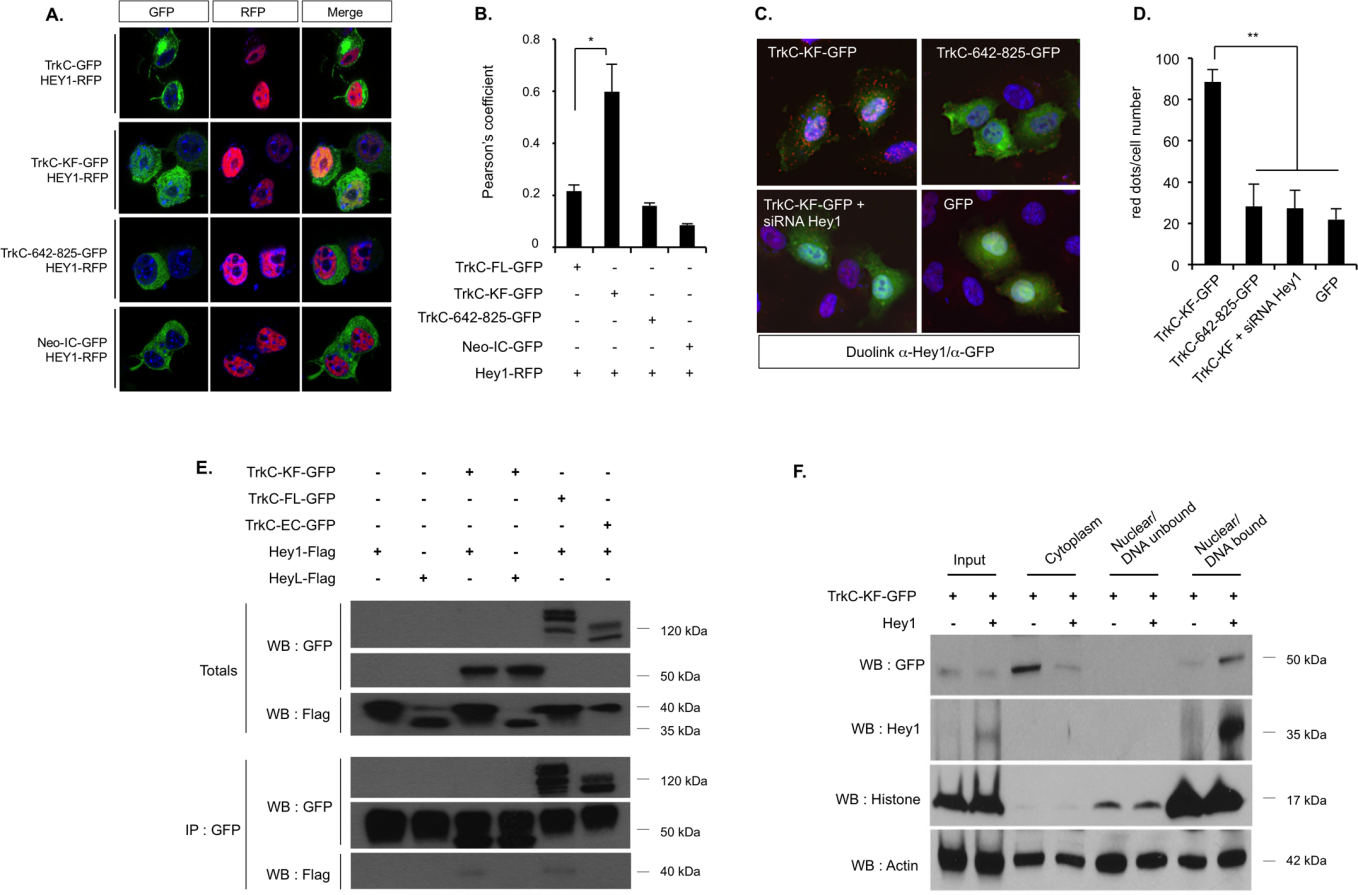
TrkC-KF associates to the transcription factor Hey1 in the nucleus, and both bind to the chromatin. (A,B) Expression of TrkC-KF-GFP and Hey1-RFP in N2A cells indicates a partial colocalization in the nucleus, as shown by confocal analysis (A) and by the associated Pearson’s coefficient (B). As controls, TrkC, TrkC-642-825, and Neo-IC (fused to GFP) were also transfected. Data represent mean ± SEM (2 independent experiments). *p < 0.05. *t* test compared to control (TrkC-GFP). (C) Proximity ligation assay (DuoLink) using an anti-Hey1 antibody (recognizing endogenous Hey1) and an anti-GFP antibody on SHEP cells transfected with TrkC-KF-GFP, TrkC-642-825-GFP, GFP, or TrkC-KF-GFP and an siRNA against Hey1: The protein–protein interactions are visualized by red fluorescent spots (Cy3). (D) Quantification of the proximity ligation assay presented in (C): Data represent mean ± SEM (4 independent fields). **p < 0.01. *t* test compared with TrkC-KF. (E) Immunoprecipitation of TrkC-KF-GFP and TrkC-642-825-GFP using an anti-GFP antibody in HEK293T transfected cells. Hey1 and HeyL are revealed by an anti-Flag WB. (F) HEK293T cells transfected with TrkC-KF-GFP and Hey1-Flag constructs were fractionated into cytoplasmic, nuclear/DNA-unbound, and nuclear/DNA-bound fractions. Actin and Histone H3 are used as loading controls. “Input” corresponds to construct expression in whole cell lysates. Underlying data can be found in S1 Data. GFP, green fluorescent protein; HEK293T, human embryonic kidney 293 T; IP, immunoprecipitation; N2A, Neruo2a; Neo-IC, intracellular fragment of Neogenin; RFP, red fluorescent protein; siRNA, small interfering RNA; TrkC, tropomyosin receptor kinase C; TrkC-FL, full-length TrkC; TrkC-KF, TrkC killer-fragment; WB, western blot.

As TrkC-KF does not seem to have a transcriptional activity, we looked for putative nuclear interacting partners in the 2-hybrid screen mentioned in Fig 1, using TrkC-KF as bait. We focused on Hey1, which was identified as a putative partner of TrkC-KF in the screen (S1B Fig). Hey1 is a transcription factor that belongs to the bHLH-Orange (bHLH-O) family of transcriptional repressors, together with Hey2 and HeyL [18]. NOTCH pathway activation increases Hey1 expression, leading to the transcriptional inhibition of downstream targets. Therefore, Hey1 is a critical effector of the NOTCH pathway, being involved in cardiac and vascular development [19]. Hey1 is mostly nuclear but has also been detected in the cytoplasm [20]. We showed by confocal microscopy that TrkC-KF-GFP colocalizes with Hey1 tagged with red fluorescent protein (Hey1-RFP) in the nucleus of N2A cells (Fig 2AB). Silencing Hey1 with a designed small interfering RNA (siRNA) was associated with a strong reduction of Hey1 mRNA (S2A Fig) or protein (S2B Fig) without affecting the level of other members of the bHLH-O transcription factor family members Hey2 and HeyL (S2A and S2C Fig). We further showed in SHEP cells that TrkC-KF-GFP and endogenous Hey1 are interacting, by using an anti-Hey1 antibody in a proximity ligation assay (Duolink) (Fig 2C and 2D). No signal of interaction was detected when TrkC 642–825 was used instead of TrkC-KF or when Hey1 was silenced with an siRNA (Fig 2C and 2D). Furthermore, TrkC-KF-GFP nuclear localization was not affected by invalidation of endogenous Hey1 with the siRNA (S2D and S2E Fig). Thus, Hey1 specifically interacts with TrkC-KF and does not seem to be required for TrkC-KF nuclear translocation.

We next confirmed by coimmunoprecipitation performed in human embryonic kidney 293 T (HEK293T) cells that Hey1 interacts with TrkC-KF-GFP and full-length TrkC (TrkC-FL-GFP) (Fig 2E). Furthermore, HeyL, a transcription factor closely related to Hey1, fails to interact with TrkC-KF of TrkC-FL, showing that TrkC specifically interacts with Hey1 only (Fig 2E).

Interestingly, when cotransfected with Hey1, TrkC-KF-GFP was markedly detected in the nucleus in the DNA-bound fraction (Fig 2F). Thus, Hey1 interaction with TrkC-KF allows their joint binding to DNA complex.

### Hey1 is a TrkC proapoptotic partner

**Fig 3:**
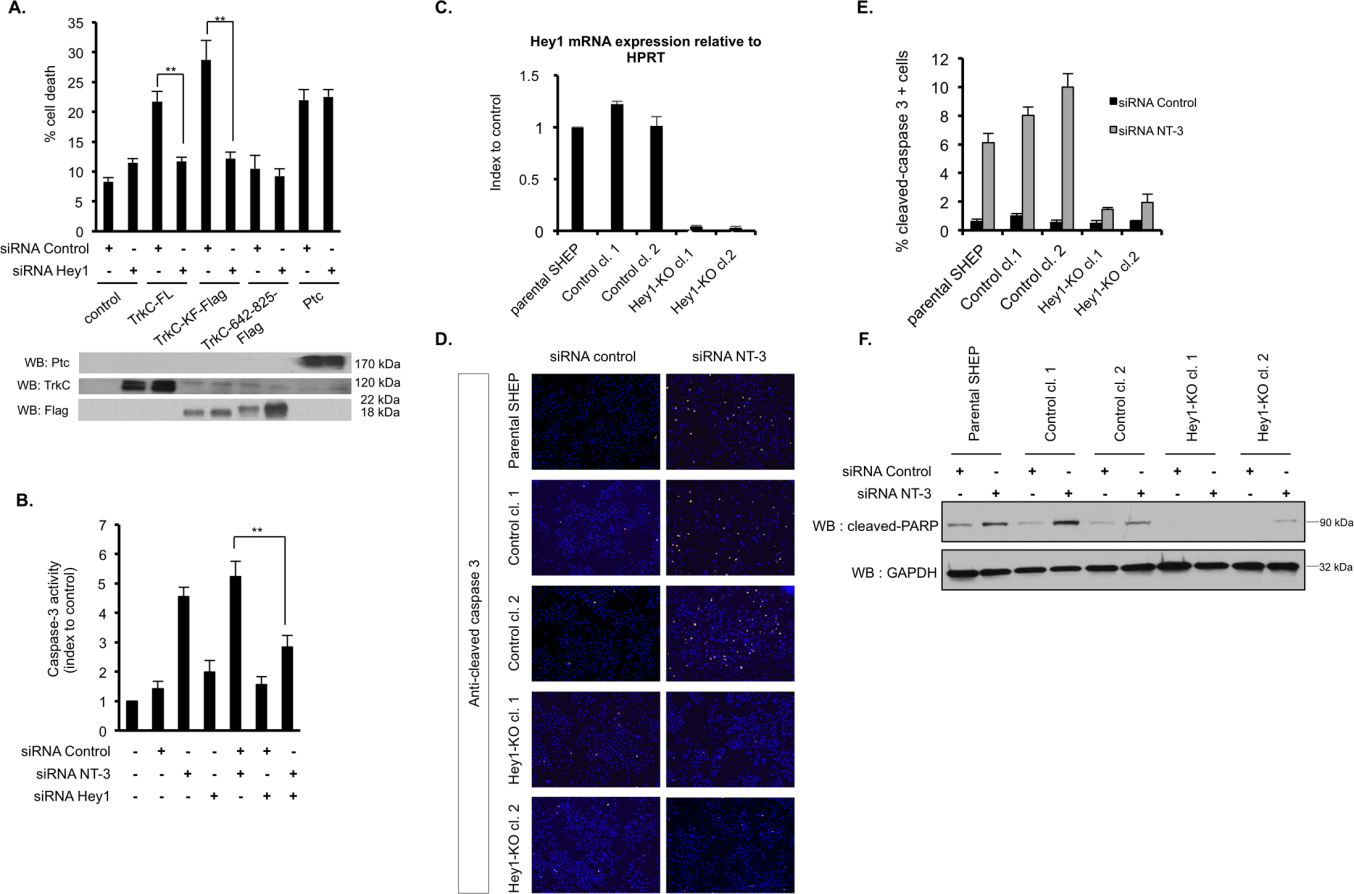
Hey1 is essential for the cell death mediated by TrkC. (A) Quantification of cell death by trypan blue exclusion in N2A cells transfected with the indicated constructs (Ptc) and an siRNA control or an siRNA targeting Hey1. Expression of each construct was assessed by WB (lower panel). Data represent mean ± SEM (*n* = 5). **p < 0.01. *t* test. (B) Caspase-3 activity assay on SHEP cells transfected with siRNA control, siRNA NT-3, and siRNA Hey1. Data represent mean ± SEM (*n* = 3) indexed to control. **p < 0.01. *t* test. (C) Hey1 mRNA expression assessed by RT-QPCR on SHEP clones isolated from SHEP cells transiently transfected with a CAS9-only-expressing vector (Control clones 1 and 2) or a CAS9- and Hey1-targeting gRNA-expressing vector (Hey1-KO clones 1 and 2). Data represent mean ± SEM (*n* = 3) relative to HPRT mRNA expression (housekeeping gene) and indexed to control. Parental SHEP are used as control. (D) Immunofluorescence staining using an anticleaved caspase-3 antibody (Cy3) performed on parental, control, and Hey1-KO SHEP cells and clones after transfection with siRNA control or siRNA NT-3. A representative picture is shown for each condition. Nuclei are stained with DAPI. (E) Quantification of the cleaved caspase-3 staining shown in (D) as a percentage of total cell number measured by DAPI staining. Data represent mean ± SEM (*n* = 3 independent fields). (F) Cleaved-PARP protein level was assessed by WB in parental, control, and Hey1-KO SHEP cells and clones after transfection with siRNA control or siRNA NT-3. GAPDH is used as a loading control. Underlying data can be found in S1 Data. CAS9, clustered regularly interspaced short palindromic repeat–associated protein 9; GAPDH, glyceraldehyde 3-phosphate dehydrogenase; gRNA, guide RNA; HPRT, hypoxanthine phosphoribosyltransferase; KO, knock-out; N2A, Neuro-2a; NT-3, neurotrophin-3; PARP, poly [ADP-ribose] polymerase; Ptc, Patched; RT-QPCR, quantitative real-time PCR; siRNA, small interfering RNA; TrkC, tropomyosin receptor kinase; TrkC-FL, full-length TrkC; TrkC-KF, TrkC killer-fragment; WB, western blot.

In a previous study, we set up conditions in order to transiently express TrkC-FL without inducing an artefactual dimerization and subsequent activation of the kinase domain, as it may be the case with overexpressed RTKs [12, 13]. In this setting, we observed in absence of NT-3 that the expression of TrkC-FL or TrkC-KF induces cell death in various cancer cell lines, including N2A NB cells [13]. We show here that silencing of Hey1 by RNA interference abrogates cell death induced by TrkC-FL or TrkC-KF in N2A cells (Fig 3A). As a control, the apoptosis induced by another dependence receptor, Patched (Ptc), is not affected by Hey1 silencing (Fig 3A). Rather than forcing TrkC expression, we investigated whether cell death induced upon NT-3 withdrawal is also dependent on Hey1. We have previously shown that SHEP cells are expressing both NT-3 and TrkC and that silencing of NT-3 is associated with TrkC-induced apoptosis in these cells [12]. We therefore silenced Hey1 in NT-3-depleted SHEP cells. As shown in Fig 3B, caspase-3 activation induced by NT-3 deprivation is similarly abrogated by cosilencing of endogenous Hey1. We had also previously demonstrated that various other NB cell lines overexpress NT-3 [12]. Among them, CLB-Ga [21] and LAN6 [22] express NT-3 within the same range as SHEP cells (S3A Fig and [12]). Despite the fact that LAN6 also expresses another Trk receptor, TrkA (S3A Fig), we demonstrate that interfering with endogenous NT-3 by RNA interference also triggers caspase-3 activity in LAN6 cells and that this caspase-3 activity can also be abrogated by cosilencing of Hey1 (S3B and S3C Fig). As p75^NTR^ has been suggested to mediate TrkC-induced apoptosis [10], we wondered whether its cosilencing could abrogate the apoptosis induced by NT-3 deprivation in SHEP cells as well as Hey1 did. As shown in S3D and S3E Fig, the caspase-3 activity measured by endogenous NT-3 invalidation is not altered upon treatment with an siRNA targeting p75^NTR^. p75^NTR^ may thus be dispensable, at least in our model, for the apoptosis triggered by TrkC via Hey1 in absence of NT-3.

In order to work with cells constitutively knock-out for Hey1, we then invalidated endogenous Hey1 in SHEP cells by clustered regularly interspaced short palindromic repeat (CRISPR)/CRISPR-associated protein 9 (CAS9) editing and obtained 2 independent clones in which Hey1 expression was fully abolished. As a control, we used clones that had undergone the same selection process but without the transfection of the guide RNA (gRNA) (Fig 3C). We labeled the various SHEP clones and parental cells with an anticleaved caspase-3 antibody after siRNA NT-3 or siRNA control treatment. Hey1 knock-out clones displayed a much-reduced staining compared to the clones still expressing Hey1 (Fig 3D and 3E). Along this line, in a WB with an anticleaved poly [ADP-ribose] polymerase (PARP) antibody, we observed that PARP cleavage (cPARP) is greatly reduced in Hey1 knock-out clones upon siRNA NT-3 treatment (Fig 3F and S3F Fig).

Altogether, these data indicate that the transcription factor Hey1 acts as a specific proapoptotic partner/effector of endogenous TrkC.

### Hey1 contributes to TrkC-dependent p53 stabilization

**Fig 4:**
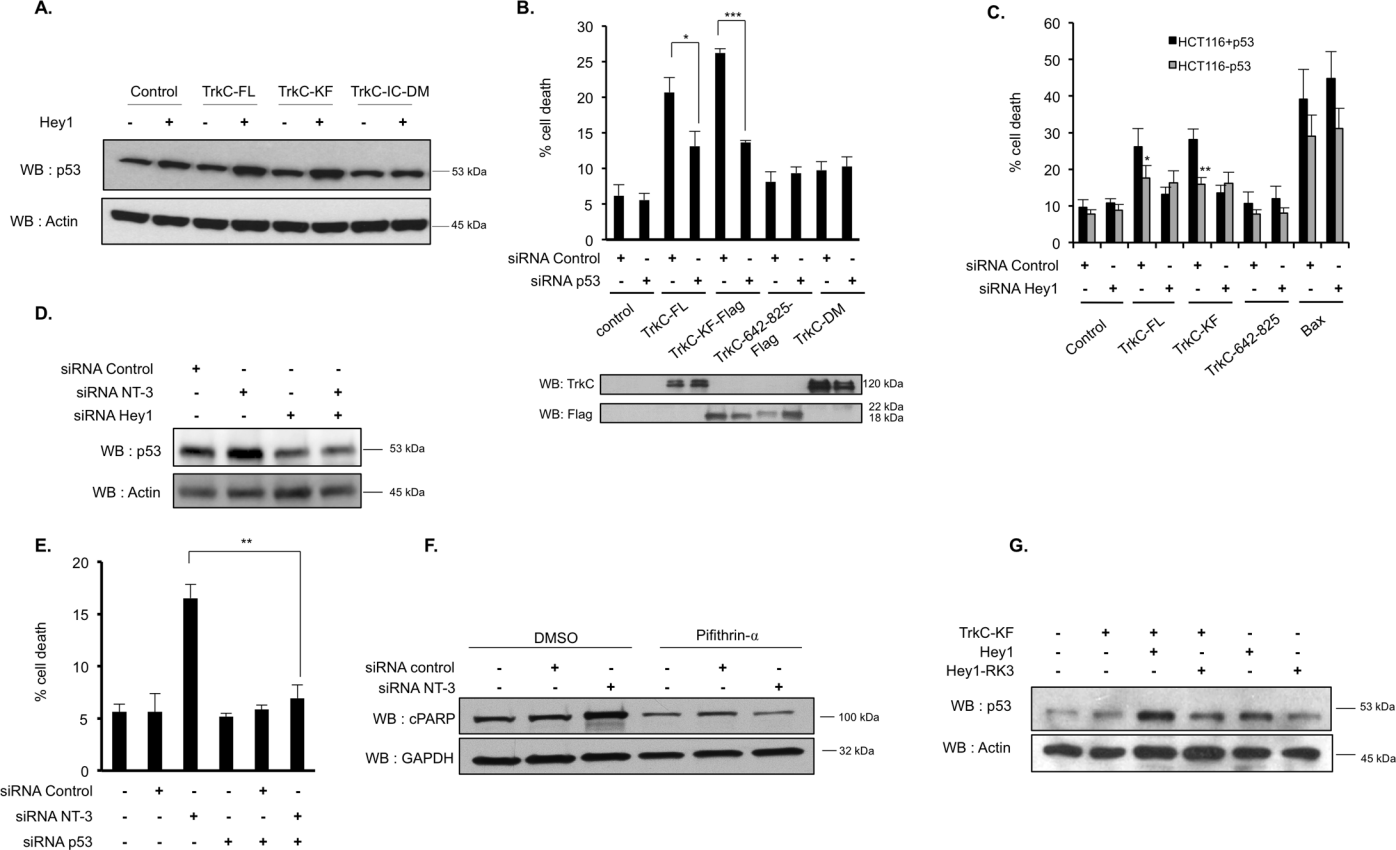
Hey1 is necessary for TrkC-KF-induced p53 stabilization. (A) p53 protein level was assessed by WB in SHEP cells transfected with the indicated constructs. This construct is not cleaved by caspase. Actin is used as a loading control. (B) Quantification of cell death by trypan blue exclusion in SHEP cells transfected with the indicated constructs and an siRNA control or an siRNA targeting p53. Expression of each construct was assessed by WB. Data represent mean ± SEM (*n* = 3). *p < 0.05, **p < 0.01. *t* test. (C) Quantification of cell death by trypan blue exclusion in WT HCT116 cells (gray bars) and HCT116 cells KO for p53 (black bars). Cells were transfected with the indicated constructs and an siRNA control or an siRNA targeting Hey1. Data represent mean ± SEM (*n* = 3). *p < 0.05, **p < 0.01. *t* test. (D) p53 protein level was assessed by WB in SHEP cells transfected with siRNA control, siRNA NT-3, and siRNA Hey1. Actin is used as a loading control. (E) Quantification of cell death by trypan blue exclusion in SHEP cells transfected with siRNA control, siRNA NT-3, and siRNA Hey1. Data represent mean ± SEM (*n* = 3). **p < 0.01. *t* test. (F) cPARP protein level was assessed by WB in SHEP cells transfected with siRNA NT-3 or siRNA control and treated either with Pifithrin-α (20 μM), p53 inhibitor, or with its vehicle, DMSO. GAPDH is used as a loading control. (G) p53 protein level was assessed by WB in SHEP cells transfected with TrkC-KF, Hey1, or Hey1-RK3 (mutation affecting Hey1 DNA binding site). Actin is used as a loading control. Underlying data can be found in S1 Data. Bax, B cell lymphoma 2–associated X; cPARP, PARP cleavage; GAPDH, Glyceraldehyde 3-phosphate dehydrogenase; KO, knock-out; NT-3, neurotrophin-3; PARP, poly [ADP-ribose] polymerase; siRNA, small interfering RNA; TrkC, tropomyosin receptor kinase C; TrkC-FL, full-length TrkC; TrkC-IC-DM, intracellular domain of TrkC mutated on D495N and D641N; TrkC-KF, TrkC killer-fragment; WB, western blot; WT, wild-type.

Of interest, Hey1 has been previously identified in a screen aimed at finding new activators of p53 [23]. This study indeed demonstrated that Hey1 stabilizes p53 by down-regulating the expression of the p53 antagonist, MDM2. Along this line, we were able to detect an increase in the amount of p53 protein in SHEP cells forced to express Hey1, and this increase was more important when Hey1 was coexpressed with TrkC-FL or TrkC-KF (Fig 4A and S4A Fig). Conversely, coexpression of Hey1 with the intracellular uncleavable form of TrkC (TrkC-IC-D495N/D641N [TrkC-IC-DM]) did not increase p53 protein amount in SHEP cells (Fig 4A and S4A Fig). In order to determine whether p53 could be involved in the apoptosis mediated by TrkC, we silenced p53 by siRNA and assessed whether TrkC-FL or TrkC-KF could still be proapoptotic. Of interest, silencing p53 abrogates TrkC-FL- or TrkC-KF-mediated apoptosis (Fig 4B and S4B Fig). We then took advantage of HCT116 cells, which have been knock-out for p53, and their parental wild-type counterparts [24]. In p53 constitutively knock-out HCT116 cells, TrkC-FL and TrkC-KF were both unable to trigger apoptosis compared to what is seen in p53 wild-type HCT116 cells (Fig 4C).

Again, rather than forcing TrkC expression, we silenced NT-3 in SHEP cells. As shown in Fig 4D and S4C Fig, silencing of NT-3 by siRNA is associated with an increased p53 protein level, whereas this is not the case when NT-3 and Hey1 are cosilenced. Similarly, cosilencing of NT-3 and p53 by RNA interference blocked NT-3 deprivation-induced apoptosis, demonstrating that p53 is necessary for TrkC apoptotic signaling (Fig 4E). As shown in S4D and S4E Fig, we also confirmed that Hey1 and p53 are necessary to unliganded TrkC–induced apoptosis in another NB cell line that expresses both NT-3 and TrkC: CLB-Ga cells (S3A Fig and [12]).

We then used a chemical inhibitor of p53-dependent transcriptional activation, pifithrin-α [25], and treated SHEP cells with an siRNA targeting NT-3 or an siRNA control. We detected an increased amount of the apoptotic cPARP fragment in siRNA NT-3-treated cells, and this effect was reversed upon treatment with pifithrin-α (Fig 4F and S4F Fig). This result suggests that p53 transcriptional activation is required to mediate TrkC-induced apoptosis.

Finally, in order to determine whether p53 stabilization is mediated by Hey1 transcription repressor function, we coexpressed TrkC-KF with a mutant version of Hey1 bearing 3 point mutations (Hey1-RK3: R50K, R54K, R62K). This triple mutation has been shown to affect Hey1 DNA-binding basic domain and consequently Hey1 transcriptional activity [26]. We observed that TrkC-KF when expressed with Hey1-RK3 is no longer able to induce p53 stabilization, supporting the view that Hey1 transcriptional activity is required for p53-dependent TrkC-KF proapoptotic activity (Fig 4G and S4G Fig).

### TrkC-KF and Hey1 cooperate in inhibiting MDM2 transcription by direct binding to *MDM2* promoter

**Fig 5:**
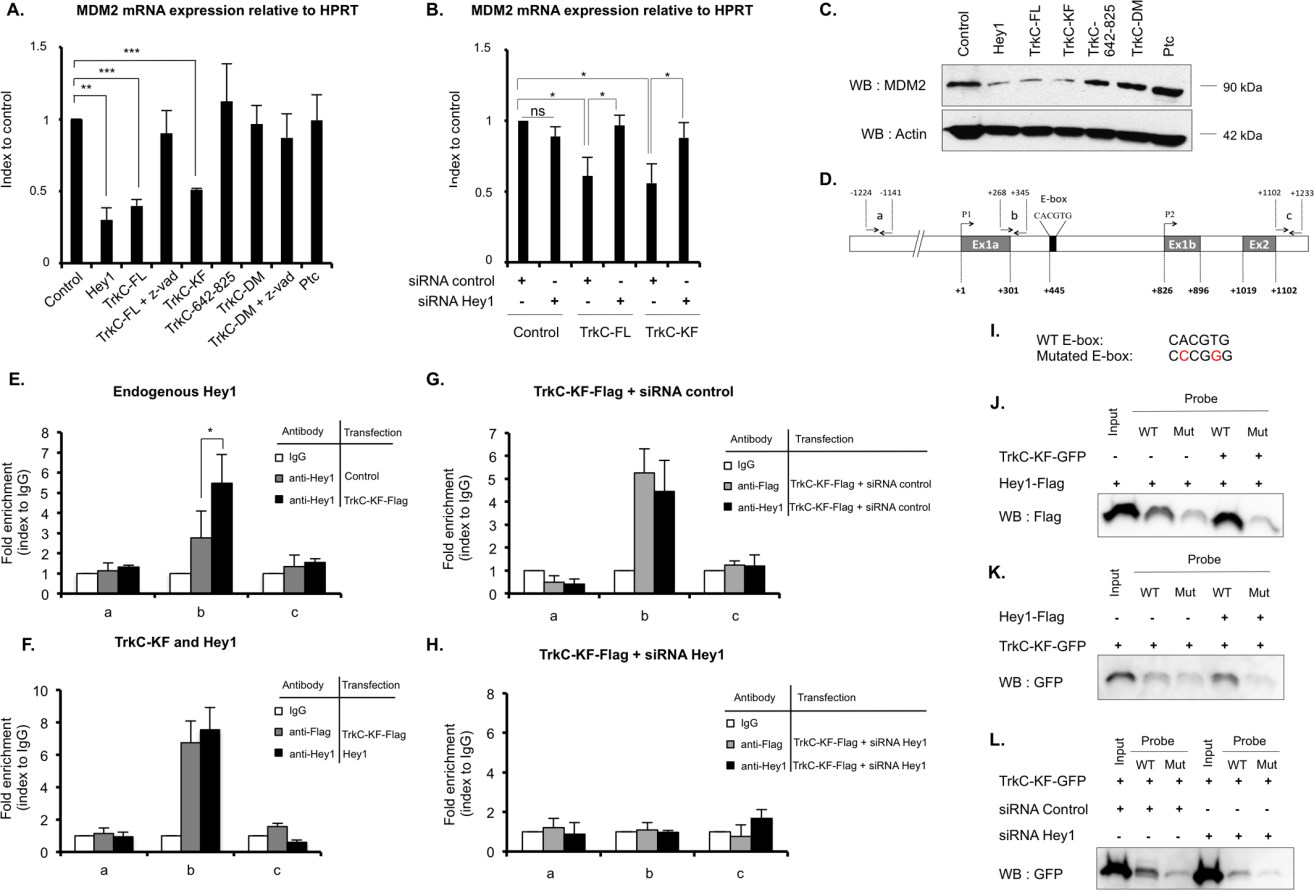
TrkC-KF and Hey1 cooperate to inhibit MDM2 transcription by direct binding on its promoter. (A) MDM2 mRNA expression was assessed by RT-QPCR on SHEP cells transfected with the indicated constructs (TrkC-DM). Data represent mean ± SEM (*n* = 3) relative to HPRT mRNA expression (housekeeping gene) and indexed to control. **p < 0.01. Two-sided Mann-Whitney test compared to control. Some cells were treated with z-vad, a general caspase inhibitor preventing TrkC cleavage. (B) MDM2 mRNA expression was assessed by RT-QPCR on SHEP cells transfected with the indicated constructs and with an siRNA control or an siRNA targeting Hey1. Data represent mean ± SEM (*n* = 3) relative to HPRT mRNA expression (housekeeping gene) and indexed to control. *p < 0.05. Two-sided Mann-Whitney test. (C) MDM2 protein level was assessed by WB in SHEP cells transfected with the indicated constructs. Actin was used as a loading control. (D) Schematic representation of *MDM2* promoter. Exons are indicated as gray boxes. An E-box contained in the regulated promoter of *MDM2* is indicated as a black box. The couples of primers a, b, and c used in the ChIP experiments described in figures (E) and (F) are also indicated. (E,F) ChIP assays using chromatin isolated from SHEP cells transfected with a control plasmid or TrkC-KF-Flag (E) or transfected with TrkC-KF-Flag or Hey1 (F). Proteins were immunoprecipitated with an anti-Flag antibody for TrkC-KF and an anti-Hey1 antibody for Hey1, as indicated. DNA sequences from *MDM2* promoter were amplified by RT-QPCR using couples of primers a (upstream control), b (binding site), and c (downstream control). Data represent mean ± SEM (*n* = 3) indexed to isotypic IgG value. *p < 0.05 *t* test. (G,H) ChIP assays using chromatin isolated from SHEP cells, transfected with TrkC-KF-Flag, and an siRNA control (G) or an siRNA targeting Hey1 (H). Proteins were immunoprecipitated with a control antibody (white bars), an anti-Flag antibody for TrkC-KF (gray bars), and an anti-Hey1 antibody for endogenous Hey1 (black bars). DNA sequences from *MDM2* promoter were amplified by RT-QPCR using couples of primers a (upstream control), b (binding site), and c (downstream control), indicated on (D). Data represent mean values ± SEM (*n* = 3) indexed to isotypic IgG value. (I) Sequences of *MDM2* promoter WT E-box and mutated E-box used in (J), (K), and (L). (J,K,L) DNA pull down assay using 80 bp double-stranded oligonucleotides (probe) corresponding to TrkC-KF and Hey1 binding site on *MDM2* promoter: WT probe (WT) contains the WT sequence of E-box; mut contains the mutated E-box indicated in (I). Biotinylated probes were incubated with nuclear lysates from SHEP cells transfected with TrkC-KF-GFP, Hey1-Flag, or both. Hey1 was revealed by an anti-Flag WB (J), TrkC-KF was revealed by an anti-GFP WB (K) and (L). “Input” corresponds to protein expression in whole nuclear lysate before incubation with the probes. (L) Biotinylated probes were incubated with nuclear lysate from SHEP cells transfected with an siRNA control or an siRNA targeting endogenous Hey1. Underlying data can be found in S1 Data. ChIP, chromatin immunoprecipitation; E-box, enhancer box; GFP, green fluorescent protein; HPRT, hypoxanthine phosphoribosyltransferase; IgG, immunoglobulin G; MDM2, mouse double minute 2 homolog; mut, mutated probe; Ptc, Patched; RT-QPCR, quantitative reverse transciption PCR; siRNA, small interfering RNA; TrkC, tropomyosin receptor kinase C; TrkC-DM, TrkC-D495N/D641N; TrkC-FL, full-length TrkC; TrkC-KF, TrkC killer-fragment; WB, western blot; WT, wild-type.

Because we (i) failed to detect any interaction between MDM2 protein and neither Hey1 nor TrkC-KF by proximity ligation assay (S5A Fig), (ii) observed that Hey1 DBD appears to be important for TrkC-KF/Hey1-mediated stabilization of p53 (Fig 4G), and (iii) identified *MDM2* in a chromatin immunoprecipitation sequence (ChIP-Seq) aimed at screening Hey1 binding sites in Hey1 overexpressing cells (Gene Expression Omnibus [GEO] accession number GSE60699 [27]), we hypothesized that Hey1/TrkC-KF may transcriptionally regulate MDM2 expression. We first measured MDM2 expression by quantitative real-time PCR (RT-QPCR) in SHEP cells transfected with various expression plasmids. As described previously by Huang and colleagues, forced expression of Hey1 is able to deregulate MDM2 expression [23]. Of interest, TrkC-FL itself is also able to deregulate MDM2 expression (Fig 5A). As a control, the TrkC 642–825 fragment, the uncleavable form of TrkC (TrkC-DM), TrkC-FL in presence of caspase inhibitor z-vad, or an unrelated overexpressed receptor Ptc does not significantly alter MDM2 expression (Fig 5A). We further assessed the importance of Hey1 in TrkC-mediated MDM2 repression. As shown in Fig 5B, silencing of endogenous Hey1 by an siRNA in SHEP cells, forced to express either TrkC-FL or TrkC-KF, restores MDM2 expression. TrkC-FL- and TrkC-KF-mediated down-regulation of MDM2 was also observed at the protein level, as shown by western blot on transfected SHEP cells (Fig 5C and S5B Fig). Again, as a control, TrkC 642–825, TrkC-DM, or Ptc had no effect on MDM2 protein level (Fig 5C and S5B Fig).

As illustrated in Fig 5D, the *MDM2* gene has 2 promoters and an enhancer box (E-box) described as a putative binding site for various transcription factors, including bHLH-O factors like Hey1 [28]. We designed various primers all along the *MDM2* promoter region and performed chromatin immunoprecipitation (ChIP) on SHEP cells expressing Flag-tagged versions of TrkC-KF and/or Hey1. ChIP with an antibody targeting endogenous Hey1 resulted in a slight enrichment of the promoter region amplified by primers located in close proximity to the E-box (Fig 5E). As a negative control, no enrichment was observed after the use of primers designed in 5′ or in 3′ of *MDM2* promoter region (Fig 5E). Interestingly, TrkC-KF favors endogenous Hey1 binding to *MDM2* promoter, as observed by the increased DNA enrichment in TrkC-KF-Flag transfected cells when compared to nontransfected cells expressing endogenous Hey1 only (Fig 5E). When SHEP cells were forced to express TrkC-KF-Flag or Hey1, a similar enrichment of the same promoter region was observed when chromatin was pulled down with either an anti-Flag antibody (targeting TrkC-KF) or an anti-Hey1 antibody (targeting Hey1) (Fig 5F). Together, these results support the view that TrkC-KF and Hey1 interact and bind to the same promoter region near the E-box of *MDM2* promoter. To more formally address this question, we silenced Hey1 in TrkC-KF-Flag-expressing SHEP cells. Silencing of Hey1 fully reversed the DNA enrichment observed, indicating that TrkC-KF binding on *MDM2* promoter is dependent on its interaction with Hey1 (Fig 5G and 5H). Finally, to assess direct binding of Hey1 and TrkC-KF to the *MDM2* promoter E-box, we proceeded to an oligonucleotide pull-down assay using biotin-labeled double-stranded oligonucleotides homologous to the promoter region spanning *MDM2* E-box. Oligonucleotides were mutated (mut) or not (WT) on the E-box sequence (Fig 5I) and incubated with lysates of SHEP cells expressing Hey1-Flag and TrkC-KF-GFP. As illustrated in Fig 5J and 5K, we could demonstrate the association of Hey1 (Fig 5J) and TrkC-KF (Fig 5K) with the oligonucleotide containing the wild-type E-box and much less with the mutated E-box oligonucleotide control. The number of bound oligonucleotides is increased when both Hey1 and TrkC-KF are expressed (Fig 5J and 5K). Conversely, silencing of endogenous Hey1 strongly inhibits TrkC-KF binding to the oligonucleotides corresponding to *MDM2* promoter (Fig 5L). These data further confirm the association of Hey1 with TrkC-KF in the promoter region spanning *MDM2* E-box. Together, these results show that TrkC-KF and Hey1 interact on *MDM2* promoter and inhibit *MDM2* transcription.

We described in a previous study the shuttling of TrkC-KF at the mitochondria by COBRA1, in which both partners activated BAX and induced MOMP, the subsequent release of cytochrome *c*, and apoptosome activation [13]. What is then the role of the nuclear pathway and p53 stabilization by TrkC-KF/Hey1 interaction? Are the mitochondrial and the nuclear pathways redundant, or are they sequentially activated? We first expressed TrkC-FL in N2A cells and invalidated Hey1 by siRNA to abrogate cell death. We observed that in this setting, transient overexpression of COBRA1 is sufficient to largely restore apoptosis (S5C Fig). Conversely, Hey1 expression does not significantly restore cell death upon COBRA1 silencing (S5D Fig). These results suggest that the nuclear Hey1/p53 pathway is acting upstream the COBRA1/BAX mitochondrial pathway.

We made the hypothesis that p53 activation may transcriptionally supply the proteins that are essential for the mitochondrial pathway triggered by TrkC. Indeed, COBRA1 promoter has been previously identified as a target of p53 in a genome-wide ChIP assay [29]. We measured COBRA1 expression by RT-QPCR and observed that invalidation of endogenous NT-3 by RNA interference (i.e., the activation of TrkC/Hey1/p53 pathway) increases the amount of COBRA1 mRNA in SHEP cells (S5E Fig). This effect was reversed upon coinvalidation of NT-3 with Hey1 or p53 (S5E Fig). This result suggested that, indeed, p53 is important to allow the expression of COBRA1.

In order to determine whether p53 is responsible for this transcriptional up-regulation of COBRA1, we identified 2 putative p53 binding sites [30] on the *COBRA1* promoter and designed various pairs of primers spanning different regions of the *COBRA1* promoter (S5F Fig). The chromatin of SHEP cells transfected with either control or TrkC-KF and Hey1 was pulled down with an anti-p53 antibody, and the region encompassing the 2 putative p53 sites was more amplified than the 5′ or 3′ region of the promoter (S5G Fig). These results suggest that p53 indeed binds to the *COBRA1* promoter and contributes to the supply of COBRA1 proteins in the cytoplasm so that a pool of TrkC-KF fragments produced by the caspase cleavage in the cytoplasm can be shuttled at the mitochondria by COBRA1 proteins.

We also showed in our previous study that TrkC-KF and COBRA1, once anchored at the mitochondrial membrane, activate BAX—but not B cell lymphoma 2 killer (BAK)—to trigger MOMP [13]. BAX is a well-characterized target of p53 [31], so we also designed pairs of primers among which 1 pair spanned the p53 binding site (S5H Fig). The chromatin region amplified by this pair of primers was greatly amplified in SHEP cells transfected with TrkC-KF and Hey1 (S5I Fig). Conversely, *BAK* promoter, another p53 target [32], was not amplified when this experiment was repeated with primers spanning the p53 binding site on the *BAK* promoter (S5J and S5K Fig). These results are consistent with the fact that the TrkC proapoptotic pathway does not require BAK but requires BAX and COBRA1.

Together, these data support the idea that the nuclear apoptotic pathway triggered by TrkC-KF with Hey1 and p53 is essential to provide the adequate number of TrkC proapoptotic partners in the cytoplasm to finally induce MOMP and apoptosome activation.

### Hey1/p53-dependent TrkC-induced apoptosis constrains tumor growth in vivo

**Fig 6:**
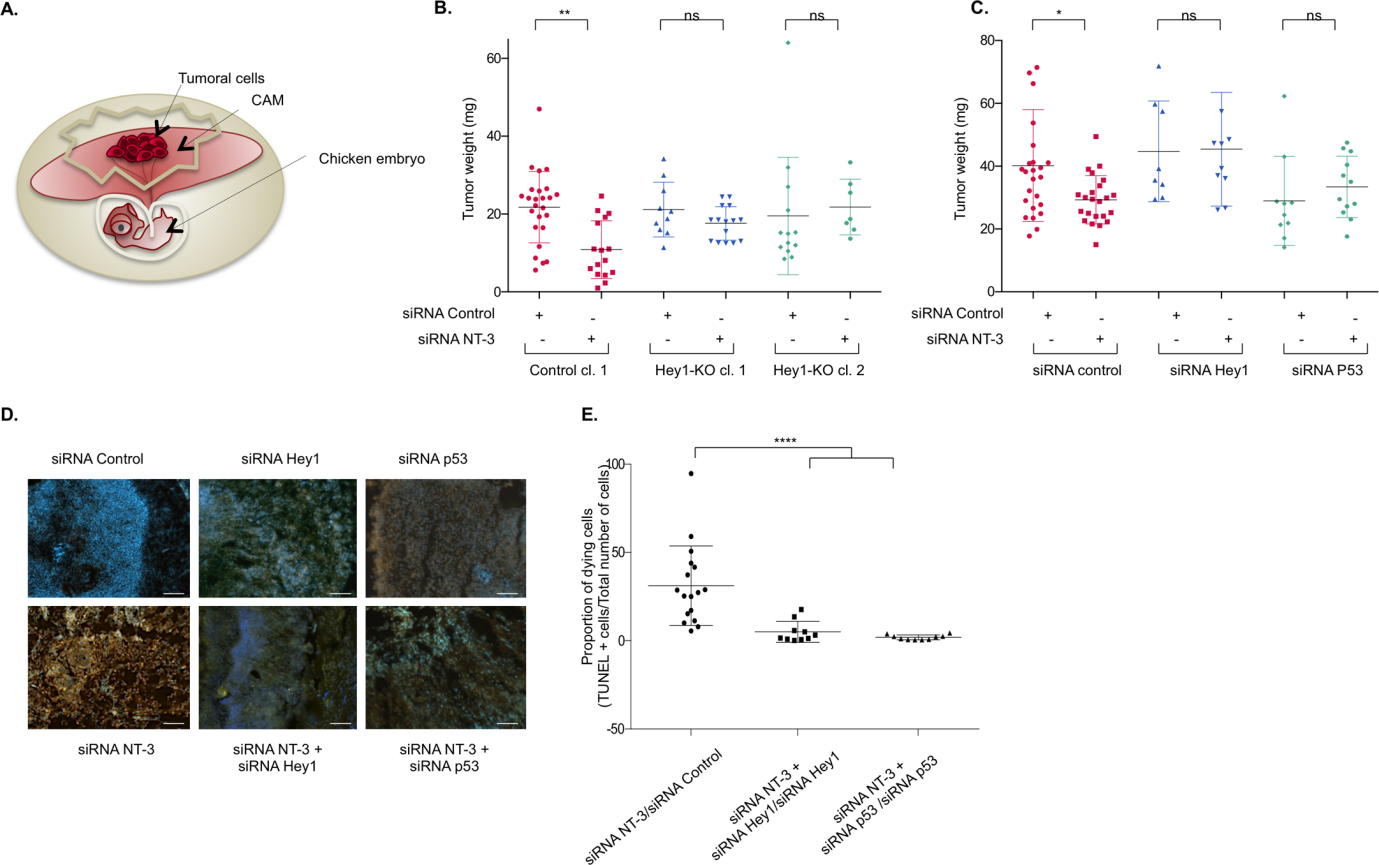
p53 and Hey1 are essential for TrkC-mediated inhibition of tumoral growth in a xenograft model of neuroblastoma. (A) Schematic representation of the experimental setting of tumor growth in the chick embryo. Tumoral cells are inoculated on the CAM of E10 chick embryo. Five d later, a primary tumor has grown and can be dissected, weighted, and immunohistologically analyzed. (B) Measures of tumor weights 5 d after inoculation of Control (cl.1) and Hey1-KO (cl.1 and cl.2) SHEP clones generated by CRISPR/CAS9 editing and transfected with siRNA control or siRNA NT-3. A one-way ANOVA with Sidak’s multiple comparison test is performed (siRNA NT-3 versus siRNA). **p < 0.01. (C) Measures of tumor weights for each condition, as indicated. A one-way ANOVA with Sidak's multiple comparison test is performed (siRNA NT-3 versus siRNA control, siRNA NT-3 + siRNA Hey1 versus siRNA Hey1, siRNA NT-3 + siRNA p53 versus siRNA p53), taking into account siRNA intrinsic toxicity of siRNA p53. *p < 0.05. (D) TUNEL (orange) and DAPI (blue) staining performed on cryosections of the dissected tumors from (C). A representative picture is shown for each condition. ×20 magnification, scale bar = 1 μm. (E) TUNEL-positive cells and DAPI-positive cells were quantified by ImageJ64 for *n* = 3–8 sections of at least 3 tumors for each condition. The ratio of TUNEL-positive cells over the total number of cells (DAPI positive) is calculated. A ratio with the value obtained in the corresponding control condition (siRNA NT-3 versus siRNA control, siRNA NT-3 + siRNA Hey1 versus siRNA Hey1, siRNA NT-3 + siRNA p53 versus siRNA p53) is calculated as indicated for each section to consider siRNA intrinsic toxicity, and the mean values ± SEM (*n* = 3) are indicated. A 2-sided Mann-Whitney *t* test is performed, ****p < 0.0001. Underlying data can be found in S1 Data. CAM, chorioallantoic membrane; CAS9, CRISPR-associated protein 9; CRISPR, clustered regularly interspaced short palindromic repeat; KO, knock-out; ns, nonsignificant; NT-3, neurotrophin-3; siRNA, small interfering RNA; TrkC, tropomyosin receptor kinase C.

We previously demonstrated that TrkC-mediated apoptosis constrains tumor growth in NB [12] and proposed that some NB cells escape from TrkC-induced apoptosis by up-regulating NT-3. We thus took advantage of an avian model in which we showed that interference with NT-3/TrkC is associated with NB growth inhibition [12, 33]. SHEP cells were inoculated on the highly vascularized chorioallantoic membrane (CAM) of E10 chicken embryos (Fig 6A). Five days later, a primary tumor was formed at the inoculation site. When we inoculated parental SHEP cells or the control clone of SHEP cells, silenced for NT-3 by RNA interference, the size and weight of the tumors were reduced in comparison with tumors generated by scramble siRNA-transfected cells (Fig 6B and 6C). We inoculated 2 independent clones knock-out for Hey1 (CRISPR/CAS9 edited as shown in Fig 3). The weight of the tumor did not vary significantly in Hey1 knock-out clones upon NT-3 invalidation by siRNA, suggesting that Hey1 is necessary for TrkC to limit tumor progression in absence of NT-3 (Fig 6B). We also observed that cosilencing of NT-3 with either Hey1 or p53 by RNA interference in SHEP cells also reverses siRNA NT-3-induced tumor suppressive effect (Fig 6C), as it had previously been demonstrated upon NT-3 and TrkC cosilencing [12]. The reduction in size of tumors formed by NT-3-silenced SHEP cells was associated with high apoptosis, as shown by TUNEL staining performed on tumor cryosections (Fig 6D and 6E). As expected, this induction of apoptosis triggered by NT-3 silencing is reversed when Hey1 or p53 is invalidated (Fig 6D and 6E). These results demonstrate in vivo that TrkC tumor suppressor activity requires Hey1 and p53.

To analyze whether silencing of the proapoptotic pathway induced by TrkC/Hey1 in absence of NT-3 could be associated with patient poor prognosis, we analyzed various transcriptomic analyses performed on human NB tumors (S6 Fig). It had previously been shown in a limited number of human samples that TrkC expression is associated with favorable outcome in NB [34] and that Hey1 expression is greatly reduced in NB when compared with benign tumors [35]. We made the same observation on a larger cohort, published by T. Wolf on the National Center for Biotechnology Information (NCBI) GEO, analyzed by Agilent-Microarray 44K (GSE45480, 649 samples) for both TrkC and Hey1 (S6A and S6B Fig). TrkC and Hey1 expression is significantly lower in aggressive stage 4 NB tumors than in stage 1–3 NB tumors. We further calculated the intergrade median of expression for NT-3, TrkC, and Hey1. We then selected 3 profiles of tumors based on the mode of action of NT-3 inhibiting the death induced by TrkC and Hey1: (i) tumors which express low levels of NT-3 (beyond the intergrade median of NT-3 expression), high levels of TrkC, and high levels of Hey1—i.e., tumors in which TrkC is prone to induce apoptosis (NT-3^low^, TrkC^high^, Hey1^high^); (ii) tumors in which the 3 genes are expressed at a low level or silenced—i.e., this death pathway is blocked (NT-3^low^, TrkC^low^, Hey1^low^); and (iii) tumors with other profiles. As shown in S6C Fig, the proportion of tumors in which the TrkC death pathway is "ON" (NT-3^low^, TrkC^high^, Hey1^high^) is high in low-grade NB1–3 tumors but decreases when the grade increases (22% in NB1–3 and 12% in NB4). Conversely, the percentage of tumors in which the death pathway is "OFF" (NT-3^low^, TrkC^low^, Hey1^low^) is limited in low-grade tumors and increases with tumor aggressiveness in NB (S6C Fig). This result is in agreement with our hypothesis that a functional TrkC proapoptotic pathway is associated with a favorable outcome, whereas the silencing of this pathway confers a selective advantage to NB tumors. We observed the same trend when analyzing 3 microarrays performed on other cohorts (L. Shi [36], O. Delattre [37], and R. Versteeg [35]). Finally, a Kaplan-Meier analysis performed on the survival data of the T. Wolf cohort indicated that tumors having a functional TrkC death pathway (Group A: NT-3^low^, TrkC^high^, Hey1^high^) are significantly associated with a better prognosis than tumors with silenced TrkC proapoptotic pathway (Group B: NT-3^low^, TrkC^low^, Hey1^low^) or other types of tumors (Group C) (S6D Fig).

Altogether, these data support the view that TrkC constrains tumor growth via Hey1- and p53-mediated apoptosis in vivo, and this proapoptotic pathway is affected in patients with high-grade tumors.

## Discussion

We previously demonstrated that, upon NT-3 deprivation, TrkC-KF is released and shuttled to the mitochondria by its proapoptotic partner, COBRA1. Once at the mitochondria, TrkC-KF and COBRA1 activate BAX and induce the MOMP and the subsequent activation of the apoptosome (S7 Fig and [13]). With this work, we decipher the complex upstream mechanisms involved in TrkC-induced cell death. TrkC-KF is translocated in the nucleus via importins and interacts there with Hey1. Hey1 and TrkC-KF interact and jointly bind to *MDM2* promoter E-box, in which TrkC-KF favors Hey1 repressor function on *MDM2* transcription. *MDM2* transcriptional inhibition promotes p53 stabilization and thus apoptosis. p53 target genes include COBRA1 [29] and BAX [31]. We show here that forced expression of TrkC-KF and Hey1 is associated with enhanced p53 binding to *COBRA1*- and *BAX*-respective promoters. Furthermore, we show that COBRA1 expression is enhanced by the activation of the TrkC/Hey1/p53 pathway. When this pathway is altered by Hey1 silencing, the supply of COBRA1 by transient transfection is sufficient to restore TrkC-induced apoptosis. Therefore, the nuclear function of TrkC-KF may not only lead to apoptosis through classic p53 effectors but also through the enhancement of the TrkC mitochondrial pathway by the transcriptional supply of its interactors. Similar fine regulation of proapoptotic protein amounts released in the cytoplasm has indeed already been described for p53 [38].

NB tumors derive from the sympathoadrenal lineage originating from the neural crest cells (NCCs) [39]. NCCs contribute to the formation of the peripheric ganglia, the sympathetic and sensory ganglia, and the medullary region of the adrenal gland. The adequate number of neuronal precursors in the forming ganglia during peripheric nervous development is tightly regulated by peaks of programmed cell death controlled by neurotrophin amounts in the close proximity of the precursors that express at their surface the corresponding neurotrophin receptors (for review [40]). This mechanism of programmed cell death is crucial during gangliogenesis. Indeed, an aberrant number of neuronal precursors in ganglia favors the development of NB, as already observed in mice bearing NB-driving mutations. As a first example, MycN is expressed by migrating NCC [41], and mice having a forced expression of MycN in the sympathetic lineage (TH-MycN) display a hyperplasia of paravertebral ganglia neuroblasts, which are resistant to NGF deprivation–induced apoptosis [42, 43]. In parallel, mice bearing a mutation identified in sporadic and familial cases of NB, anaplastic lymphoma kinase F1178L (ALK^F1178L^), present a higher number of sympathetic neuroblasts per ganglion than wild-type mice [44]. These studies illustrate the crucial need to clearly identify the actors, which define the adequate number of precursors in the peripheric ganglia. It has been well established that NT-3 and TrkC control the adequate number of precursors in the developing sensory ganglia [45]. We have shown in primary sensory neurons and in the chick embryo model that part of the apoptosis occurring upon NT-3 deprivation during neurodevelopment is actively triggered by TrkC itself [9] via COBRA1 [13]. Interestingly, Kessler and collaborators observed that the constitutive up-regulation of Hey1 expression in mutant mice results in a significant loss of TrkC positive sensory neurons. Conversely, Hey1 mutant mice display an increased number of TrkC-positive sensory neurons [46]. These observations are consistent with the fact that TrkC may trigger apoptosis via Hey1 in supernumerary neurons in a setting where the amount of NT-3 is limited. We decipher in this study the mechanisms that may underlie this process.

Along this line, Barde and collaborators demonstrated in murine models that TrkA and TrkC constrain the adequate number of peripheric neurons during development by actively triggering apoptosis when deprived of their respective ligands, NGF and NT-3. Conversely, TrkB has no proapoptotic activity in this context [10]. Interestingly, TrkA and TrkC expression have long been associated with regressing NB tumors, whereas TrkB expression is a marker of poor prognosis [2]. We have demonstrated here and in a previous study [12] that TrkC proapoptotic activity controls NB tumor progression. It would be of interest to determine whether TrkA and TrkC proapoptotic activity also controls NB tumor initiation in eliminating supernumerary neuroblasts or neurons in the peripheric ganglia.

In their study, Barde and colleagues suggested that p75^NTR^ intracellular domain mediated TrkA and TrkC proapoptotic activity [10]. In our N2A cellular model, p75^NTR^ is not required by TrkC to trigger apoptosis. Moreover, we were able to trigger apoptosis in 3 independent NB cell lines (SHEP, LAN6, and CLB-Ga) displaying various patterns of expression of neurotrophins and their receptors. Further investigations would be needed to investigate the putative interactions between neurotrophin receptors in the control of NB tumor progression.

With this work, we have confirmed in vivo that the TrkC/Hey1/p53 proapoptotic pathway indeed limits NB tumor growth. p53 and its inhibitor MDM2 have been particularly studied in NB (for review [47]). However, while mutations in p53 are generally considered to affect half of human adult cancers, pediatric cancers are characterized by the lack of p53 mutations [48–50]. More specifically, in NB, p53 is mutated in less than 1% of the tumors at diagnosis [51]. Tumors with wild-type p53 probably rely on other mechanisms to inactivate p53, and it is thus of interest to note that in pediatric tumors, and more specifically in NB, MDM2 is frequently up-regulated [52]. In the present study, we analyzed the transcriptomic public data sets available and showed that the silencing of TrkC proapoptotic pathway (NT-3^low^, TrkC^low^, HEY^low^) is also associated with poor patient outcome (S6 Fig). In parallel, TrkC expression has been shown to be epigenetically controlled in various cancers [4, 6]. It is thus tempting to investigate whether reactivating TrkC proapoptotic activity in these patients with p53 wild-type tumors may constitute an interesting therapeutic strategy.

## Materials and methods

### Cell culture, transfection, and treatment

N2A, SHEP, and HEK293T were described previously [13]. WT HCT116 and p53-KO HCT116 were kindly provided by B. Vogelstein (Ludwig Center at Johns Hopkins, Baltimore, MD) [24]. SHEP, LAN6, and CLB-Ga were kindly provided by V. Combaret (CRCL, Lyon)[12]. N2A cells were grown in DMEM/F-12, GlutaMAX (Life Technologies), supplemented with 10% FBS (Lonza); SHEP, LAN6, and CLB-Ga cells were grown in RPMI1640, GlutaMAX (Life Technologies), supplemented with 10% FBS (Lonza); and HEK293T, WT HCT116, and p53-KO HCT116 cells were grown in DMEM (Life Technologies), supplemented with 10% FBS (Lonza). The plasmid constructs and siRNA were transfected using JetPrime (PolyPlus) for cell death assays and Lipofectamine RNAiMAX (Life Technologies) for RT-QPCR assays following manufacturer’s instructions. Caspase activity was inhibited in SHEP cells by treatment with 20 μM of z-VAD-fmk (Merck-Millipore), a general caspase inhibitor. The pan-importins inhibitor Ivermectin (Sigma I8898) was added to the cells at a final concentration of 10 μM 2 h before cell collection. Pifithrin-α (Sigma P4359), p53 inhibitor was used at a concentration of 20 μM for 30 h.

### Plasmid construction

The plasmids encoding full-length TrkC, TrkC-KF, TrkC-642-825, TrkC495-825, TrkC-DM (TrkC-D495N/D641N), Ptc, DCC-IC, and Neogenin IC GFP were described elsewhere [9, 16]. The plasmids encoding Hey1, HeyL, and Hey1-RK3 were a kind gift of M. Gessler (University of Wuerzburg, Germany) [26]. TrkC-KF-ΔNLS 1 and 2 were generated by site-directed mutagenesis using QuickChange kit (Stratagene) and the following primers: TrkC-KF-ΔNLS1 forward: 5′-CATATGTTCAACACATCGCCGCCGCCGACATCGTGTTGAAG-3′, reverse: 5′-CTTCAACACGATGTCGGCGGCGGCGATGTGTTGAACATATG-3′. TrkC-KF-ΔNLS1/2 forward: 5′-GAGAGACATCGTGTTGGCCGCCGCCGCCGGTGAGGGAGCCTTT-3′, reverse: 5′-CAAAGGCTCCCTCACCGGCGGCGGCGGCCAACACGATGTCTCT-3′.

The plasmid encoding the sgRNA targeting Hey1 (TGACGCGCACGCCCTTGCTA) cloned into the pSPCAS9 BB-2A-GFP (PX458) was generated by GenScript.

### siRNA and shRNA

siRNAs were purchased from Sigma-Aldrich for siRNA control (siRNA Universal Negative Control #2 SIC002_10Nmol), siRNA huHey1 (NM_001040708; SASI_Hs02_00309099), siRNA hup53 (NM_000546; SASI_HS02_00302766), siRNA huCOBRA1 (NM_015456; SASI_hs01_00236976), siRNA muCOBRA1 (SASI_Mm01_00110121), and from Santa-Cruz for siRNA control (sc-37007), siRNA mHey1 (sc-42126), siRNA huNGFR p75 (sc-36057), and siRNA huNT-3 (sc-42125).

### Two-hybrid screen

The 2-hybrid screen was performed by Hybrigenics (Paris, France) using the Mouse Embryo Brain RP2 library as a prey and pB27 (N-LexA-bait-C fusion) and pB66 (N-GAL4-bait-C fusion) vectors. TrkC-KF construct was used as bait.

### Cell death assays

N2A and SHEP cells were transfected with the indicated constructs: After 4 h, the medium was replaced by medium without serum for 24 h to 72 h. Caspase-3 activity was measured as described in [12], using the Ac-DEVD-AFC substrate assay (Biovision, K105-400). Cell death percentages were assessed by trypan blue exclusion, as described in [13].

### Immunoprecipitation

HEK293T cells were lysed in 50 mM HEPES pH 7.6, 125 mM NaCl, 5 mM EDTA, and 0.1% NP-40 in the presence of protease inhibitors and were further incubated with an anti-GFP antibody (A11122, Life Technologies) and then with protein G sepharose (Sigma Aldrich) to pull down proteins of interest. Western blots were performed using an anti-Flag antibody (F3165, Sigma Aldrich).

### Western blotting

HEK293T, SHEP, and N2A cells were lysed in 50 mM HEPES pH 7.6, 125 mM NaCl, 5 mM EDTA, and 0.1% NP-40 in the presence of proteases inhibitors. For p53 western blots, SHEP cells were lysed in 50 mM Tris–HCl pH 7.5, 100 mM NaCl, 10% glycerol, 0.1% NP-40, and 0.2 mM EDTA in the presence of proteases inhibitors. Western blots were quantified using the ImageJ64 software.

### Antibodies

We used the following antibodies: anti-GFP (A11122, Life technologies), anti-GAPDH (sc-25778, Santa Cruz), anti-Histone (07–449, Millipore), anti-Flag (F3165, Sigma), anti-Actin (MAB1501R, Chemicon), anti-Hey1 (anti-HRT1, sc-16424, Santa Cruz), anti-p53 (SAPU, [53]), anti-Ptc (sc-6149, Santa Cruz), anti-TrkC (AF1404, R&D), anti-MDM2 (VMA00406, BioRad), anti-COBRA1 (F7E4, GeneTex), anti-BAX (sc-526, Tebu Bio), anti-BAK (G-23, Santa Cruz), anti-NT-3 (sc-547, Santa Cruz), and anticleaved PARP (9541T, Cell Signaling). The following antibodies were used for proximity ligation assays (DuoLink): anti-GFP (TP401, Biolabs), anti-Flag (F3165, Sigma), anti-Hey1 (anti-HRT1, sc-16424, Santa Cruz), anti-p53 (sc-126, Santa Cruz), and anti-importins (I1784, Sigma Aldrich).

### Immunofluorescence and immunohistochemistry

N2A, SHEP, LAN6, and CLB-Ga cells were cultured on coverslips, transfected with indicated plasmids using JetPrime, and then fixed 20 min in 4% paraformaldehyde and permeabilized in PBS/0.2% Triton. Nuclei were stained using DAPI. Images were obtained by confocal microscopy and analyzed using ImageJ64. For cleaved caspase-3 staining, after permeabilization, SHEP, LAN6, and CLB-Ga cells were incubated in blocking solution (PBS-BSA2%-normal serum 2%) for 1 h before incubation overnight with anticleaved caspase-3 antibody (9661, Cell Signaling) diluted to 1:1,000 in PBS. After incubation with secondary antibody (Alexa Fluor Donkey anti-Rabbit IgG 555, Invitrogen A31572) diluted to 1:2,000 in PBS for 1 h, slides were mounted in DAPI-fluoromount G (17984–24, EMS) and imaged using a Zeiss AxioImager microscope. Quantification was performed using ImageJ64.

### Subcellular fractionation

SHEP cells were transfected using JetPrime (PolyPlus). When indicated, cells were treated with Ivermectin (Sigma I8898) 10 μM 2 h before collection. After 4 h, the medium was replaced with medium without serum. 24 h after transfection, cells were harvested, and nuclei were isolated from cytoplasm using the Nuclei Pure Prep Isolation kit (Sigma Aldrich). Input, cytoplasmic, and nuclear fractions were analyzed by western blot, with GAPDH as cytoplasmic marker and Histone H3 as nuclear marker. HEK293T cells were transfected with TrkC-KF-GFP and Hey1 using JetPrime. After 4 h, the medium was replaced with medium without serum. Twenty-four h after transfection, cells were harvested, and cytoplasmic, DNA-bound, and DNA-unbound fractions were separated using the Subcellular Protein Fractionation Kit for Cultured Cells (ThermoFisher Scientific). Fractions were analyzed by WB, using Histone as nuclear marker and actin as loading control.

### Proximity ligation assays (DuoLink)

To assay protein interactions in cells by fluorescence, the DuoLink PLA kit was used (Sigma Aldrich). Briefly, cells were cultured on coverslips and then fixed in 4% PFA for 30 min and washed using PBS/7.5% glycine for 5 min. Cells were then permeabilized in PBS/0.2% Triton and incubated in a blocking solution for 30 min (PBS/2% BSA). After an overnight incubation with the primary antibodies, cells were incubated with Plus and Minus PLA probes. The probes were ligated and amplified using the Duolink In Situ Detection Reagents Orange (Sigma Aldrich). After several washes with the Duolink In Situ Wash Buffers for Fluorescence (Sigma Aldrich), nuclei were stained using DAPI, and coverslips were mounted in fluoromount.

The analysis was made by fluorescence microscopy, and signal quantification was assessed using the ImageJ64 software to count the number of red fluorescence spots compared to total cell number (assessed using DAPI staining).

### RNA isolation and RT-PCR RT-QPCR

To assay mRNA expression, total RNA was extracted from cells using the Nucleospin RNAII kit (Macherey-Nagel). One microgram of RNA was reverse-transcribed using the iScript cDNA Synthesis Kit (Bio-Rad). RT-QPCR was performed using a Light-Cycler 480 (Roche Applied Science) and the FastStart TaqMan Probe Master Mix (Roche Applied Science). The primers and probes (Universal Probe Library, Roche Applied Science) used are indicated on S1 Table.

### Luciferase assay

To assay TrkC-KF transcriptional activity, SHEP cells were transiently transfected with the indicated constructs fused to Gal4 DBD, a plasmid containing the firefly luciferase gene under the UAS-Gal4 control, and a plasmid coding for the Renilla luciferase gene under the CMV promoter as a control. To assess Firefly and Renilla luciferase activities, Dual-Luciferase Reporter Assay System was used following manufacturer’s instructions (Promega). Data represent Firefly value over Renilla value, indexed to control (Gal4).

### Generation of SHEP Hey1-KO clones using CRISPR/CAS9 editing

SHEP cells were transiently transfected with the plasmid encoding SpCAS9, Hey1-targeted gRNA, and GFP (Genscript, target sequence: GATAACGCGCAACTTCTGCC) using JetPrime. Two d after transfection, GFP-positive cells were sorted as single cells in 96-well plates for clonal selection. Hey1 mRNA expression level was measured by RT-QPCR for all obtained clones, and 2 clones with significant decrease in Hey1 mRNA level compared to the parental SHEP cell line were selected for further analysis. Editing of the *Hey1* gene was confirmed by sequencing for both clones. Control clones were obtained using a plasmid encoding SpCAS9 and GFP (Addgene). GFP-positive sorted clones were analyzed by RT-QPCR to confirm no change in Hey1 mRNA expression level as compared to the parental SHEP cell line, and 2 of them were selected for further analysis.

### ChIP

SHEP cells transfected with the indicated plasmids were incubated with 1% formaldehyde for cross-link: Reaction was stopped by the addition of 125 mM glycine. Cells were scraped in swelling buffer (25 mM Hepes pH 7.9, 1.5 mM MgCl2, 10 mM KCl, and 0.1% NP-40), and nuclei were isolated using dounce homogenizer. After centrifugation, nuclei were resuspended in sonication buffer (50 mM Hepes pH 7.9, 140 mM NaCl, 1 mM EDTA, 1% Triton X-100, 0.1% Nadeoxycholate, and 0.1% SDS) and sonicated to obtain chromatin fragments of 400 bp to 600 bp size. Chromatin was then incubated with primary antibodies or isotypic IgG (Sigma Aldrich) overnight: anti-Flag (F3165, Sigma), anti-Hey1 (anti-HRT1, sc-16424, Santa Cruz), and anti-p53 (sc-126, Santa Cruz). Complexes were pulled down using protein G sepharose (Sigma Aldrich). After washes, immune complexes were eluted, and cross-linking was reversed at 65°C. Eluates were incubated with RNase A and proteinase K; then, DNA was recovered by phenol-chloroform extraction. DNA fragments were analyzed by RT-QPCR using a Light-Cycler 480 (Roche Applied Science) and the FastStart TaqMan Probe Master Mix (Roche Applied Science). The primers and probes (Universal Probe Library, Roche Applied Science) used are indicated on S2 Table.

### DNA pull-down assay

To assay Hey1 and TrkC-KF ability to bind *MDM2* promoter, SHEP cells were transiently transfected with Hey1-Flag and TrkC-KF-GFP-expressing plasmids: Cells were resuspended in hypotonic buffer (10 mM HEPES, pH 7.9, 1.5 mM MgCl2, 10 mM KCl, 0.5 mM dithiothreitol with protease inhibitors [Roche]) and incubated on ice for 10 min. Nuclei were isolated by centrifugation, resuspended in RIPA buffer supplemented with complete proteases inhibitors, and incubated on a rotating wheel at 4°C for 20 min to obtain a nuclear proteic extract. Meanwhile, biotinylated oligonucleotides corresponding to Hey1 and TrkC-KF binding sites on the *MDM2* promoter, and containing either a WT E-box (CACGTG) or a mutated E-box (CCCGGG), were annealed to form double-stranded oligonucleotides of 80 bp size. For WT E-box, the forward oligonucleotide is 5′-biotinylated with the following 5′–3′ sequence: gggggctcggggcgcggggcgcggggcatgggg**cacgtg**gctttgcggaggttttgttggactggggctaggcagtcgcc. WT E-box reverse oligonucleotide: ggcgactgcctagccccagtccaacaaaacctccgcaaagccacgtgccccatgccccgcgccccgcgccccgagccccc.

For mut E-box, the forward oligonucleotide is 5′-biotinylated with the following 5′–3′ sequence: gggggctcggggcgcggggcgcggggcatgggg**cccggg**gctttgcggaggttttgttggactggggctaggcagtcgcc. Mut E-box reverse oligonucleotide: ggcgactgcctagccccagtccaacaaaacctccgcaaagccccgggccccatgccccgcgccccgcgccccgagccccc.

Three micrograms of double-stranded biotinylated oligonucleotides were incubated with 300 μg of nuclear protein extract for 2 h at 4°C. Complexes were pulled down using 50 μL of streptavidin-agarose beads (Sigma Aldrich) incubated for 1 h at 4°C. The protein-DNA-streptavidin-agarose complex was washed 3 times with RIPA buffer and loaded onto an SDS gel. Detection of Hey1-Flag and TrkC-KF-GFP proteins was performed by western blot as described in [13].

### Xenografts in ovo

SHEP cells were transfected with jet prime and siRNA NT-3 (100 nM) and/or siRNA Hey1 or sip53 (50 nM) 24 h before inoculation. Five million cells were suspended in 25 μl complete medium and 25 μl matrigel (Corning 356231) and seeded on 10-d-old (E10) chick CAM. On day 15, tumors were resected and weighted. To monitor apoptosis on primary tumors, they were fixed on 4% PFA, cryoprotected by overnight treatment with 30% sucrose, and embedded in Cryomount (Histolab). TUNEL staining was performed on tumor cryostat sections (Roche Diagnostics), and nuclei were stained with DAPI. Three to eight sections were analyzed at ×20 magnification for at least 3 tumors for each condition. TUNEL and DAPI positive cells were counted by ImageJ64 software.

### Transcriptomic data sets analysis

The expression values analyzed here are publically available in GEO database (http://www.ncbi.nlm.nih.gov/geo/). T. Wolf cohort (GSE45480[54]) analysis was performed with Agilent-020382 Human Custom Microarray 44k (GPL16876); the following data set probes were used: NT-3 (NTF3) UKv4_A_23_P360797, TrkC (NTRK3) UKv4_A_23_P205900, UKv4_A_23_P88538, and Hey1 UKv4_A_32_P83845. In this study, for TrkC and Hey1 expression values, a mean of the values obtained with the various probes was calculated. Kaplan-Meier analysis was performed in R2: Genomics Analysis and Visualization Platform (http://r2.amc.nl). The *p*-value is calculated to determine the optimal cutoff and is finally corrected by Bonferoni as described in [55]. A new grouping variable was made on the basis of NT-3, TrkC, and Hey1 as described in the main text.

### Statistical treatment of the data

Number of experiments and statistical tests used is indicated in figure legends. Statistical treatment of the data was performed with Prism 6.0e (GraphPad) and BiostaTGV online statistical software (http://marne.u707.jussieu.fr/biostatgv/).

## Supporting information

S1 Data Raw data of histograms. (XLSX)

S1 Table Primers and probes used for RT-QPCR. RT-QPCR was performed using the TaqMan technique, requiring the indicated probes (Universal Probe Library, Roche Applied Science). RT-QPCR, quantitative real-time PCR.
(XLS)

S2 Table Primers and probes used for ChIP. RT-QPCR was performed using the TaqMan technique, requiring the indicated probes (Universal Probe Library, Roche Applied Science). ChIP, chromatin immunoprecipitation; RT-QPCR, quantitative real-time PCR.
(XLS)

S1 Fig TrkC-KF is translocated to the nucleus by importins and has no intrinsic transcriptional activity per se. (A) SHEP cells transfected with either control plasmid, TrkC-KF-Flag, TrkC-FL, or Neo-IC were fractionated into cytoplasmic (Cytoplasm, marker: GAPDH) and nuclear (Nucleus, marker: histone) fractions. Input corresponds to the construct expression in whole cell lysates. (B) Candidate partners for TrkC-KF obtained in the 2-hybrid screen assay. (C) Quantification of the western blots presented in (Fig 1E): Signal of the anti-GFP western blot is compared to GAPDH signal (for the input and cytoplasmic fraction) and Histone H3 signal (for the nuclear fraction). Data represent values indexed to control (TrkC-KF). (D) Quantification of the western blots presented in (Fig 1G): Signal of the anti-GFP western blot is compared to GAPDH signal (for the input and cytoplasmic fraction) and Histone H3 signal (for the nuclear fraction). Data represent values indexed to control (TrkC-KF). (E) IP of TrkC-KF-GFP and TrkC-KF-ΔNLS1/2-GFP using an anti-GFP antibody in HEK293T-transfected cells. COBRA1 is tagged with a Flag epitope. Neo-IC-GFP is used as unrelated negative control. (F) Gal4, DCC-IC, TrkC-KF, and TrkC-495-825 mRNA expression were assessed by RT-QPCR to verify the expression of constructs used in the luciferase assay presented in Fig 1I. Data represent values (arbitrary units) relative to HPRT mRNA expression (housekeeping gene). Underlying data can be found in S1 Data. COBRA1, cofactor of breast cancer 1; DCC-IC, deleted in colorectal cancer intracellular domain; GAPDH, glyceraldehyde 3-phosphate dehydrogenase; GFP, green fluorescent protein; HEK293T, human embryonic kidney 293 T; HPRT, hypoxanthine phosphoribosyltransferase; IP, immunoprecipitation; KPNA4, karyopherin alpha 4; Neo-IC-GFP, Neogenin intracellular domain tagged with GFP; NLS, nuclear localization sequence; RT-QPCR, quantitative real-time PCR; TrkC, tropomyosin receptor kinase C; TrkC-FL, full-length TrkC; TrkC-KF, TrkC killer-fragment.
(TIF)

S2 Fig TrkC-KF associates specifically to the transcription factor Hey1 in the nucleus. (A) Mouse Hey1, Hey2, and HeyL mRNA expression were assessed in N2A cells transfected with an siRNA control or an siRNA targeting Hey1. Data represent values (arbitrary units) relative to HPRT mRNA expression (housekeeping gene). (B) Hey1 expression was assessed by western blot in N2A cells transfected with a Hey1-Flag expression construct and an siRNA control or an siRNA Hey1 at 2 different concentrations (20 nM and 30 nM). GAPDH is used as a loading control. (C) Hey2 and HeyL expression was assessed by western blot in N2A cells transfected with Hey2-Flag and HeyL-Flag constructs and an siRNA control or an siRNA targeting Hey1 at 30 nM. Actin is used as a loading control. (D,E) Expression of TrkC-KF-GFP in N2A cells, transfected with an siRNA control or an siRNA targeting Hey1, indicates a partial localization in the nucleus, as shown by confocal analysis (A) and by the associated Pearson’s coefficient (B), in presence or absence of Hey1. Data represent mean ± SEM (3 independent fields). *t* test compared to control (TrkC-GFP + siRNA control). Underlying data can be found in S1 Data. GAPDH, glyceraldehyde 3-phosphate dehydrogenase; GFP, green fluorescent protein; HPRT, hypoxanthine phosphoribosyltransferase; N2A, Neuro2a; ns, nonsignificant; siRNA, small interfering RNA; TrkC, tropomyosin receptor kinase C; TrkC-KF, TrkC killer-fragment.
(TIF)

S3 Fig Hey1 is essential for the cell death mediated by TrkC. (A) TrkA, TrkB, TrkC, NGF, BDNF, and NT-3 mRNA expression was assessed by RT-QPCR on CLB-Ga, LAN6, and SHEP cells relative to HPRT mRNA expression (housekeeping gene). A representative experiment is shown. (B) Immunofluorescence staining using Cy3 performed on LAN6 cells transfected or with the indicated siRNA. A representative picture is shown for each condition. Nuclei are stained with DAPI. (C) Quantification of the Cy3 staining shown in (B) as a percentage of total cell number measured by DAPI staining. Data represent mean ± SEM (*n* = 3 independent fields). (D) Caspase-3 activity assay on SHEP cells transfected with siRNA control, siRNA NT-3, and siRNA p75^NTR^ (p75). Data represent mean ± SEM (*n* = 3) indexed to control. **p < 0.01. *t* test. (E) p75^NTR^ expression was assessed by western blot in N2A cells transfected with a p75^NTR^ expression construct and an siRNA control or an siRNA p75^NTR^ at 2 different concentrations (50 nM and 100 nM). Actin is used as a loading control. (F) Quantification of the western blot presented in (Fig 3F): signal of the anticleaved PARP western blot is compared to anti-GAPDH signal. Data represent values indexed to control (parental SHEP transfected with siRNA control). Underlying data can be found in S1 Data. BDNF, brain-derived neurotrophic factor; Cy3, anticleaved caspase-3 antibody; GAPDH, glyceraldehyde 3-phosphate dehydrogenase; HPRT, hypoxanthine phosphoribosyltransferase; NGF, nerve growth factor; N2A, Neuro2a; NT-3, neurotrophin-3; p75^NTR^; neurotrophin receptor p75; PARP, poly [ADP-ribose] polymerase; RT-QPCR, quantitative real-time PCR; siRNA, small interfering RNA; TrkC, tropomyosin receptor kinase C.
(TIF)

S4 Fig Hey1 is necessary for TrkC-KF-induced p53 stabilization. (A) Quantification of the western blot presented in (Fig 4A), which has been reproduced and quantified 3 times: Signal of the anti-p53 western blot is compared to anti-Actin signal. Data represent values indexed to control, mean ± SEM (*n* = 3). *p < 0.05, **p < 0.01. *t* test. (B) p53 expression was assessed by western blot in SHEP cells transfected with an siRNA control and an siRNA p53 at 100 nM. Actin is used as a loading control. (C) Quantification of the western blots presented in (Fig 4D): Signal of the anti-p53 western blot is compared to anti-Actin signal. Data represent values indexed to siRNA control. (D) Immunofluorescence staining using Cy3 performed on CLB-Ga cells transfected or with the indicated siRNA. A representative picture is shown for each condition. Nuclei are stained with DAPI. (E) Quantification of Cy3 staining shown in (D) as a percentage of total cell number measured by DAPI staining. Data represent mean ± SEM (*n* = 3 independent fields). *p < 0.05, **p < 0.01. *t* test. (F) Quantification of the western blot presented in (Fig 4F): Signal of the anti-p53 western blot is compared to anti-Actin signal. Data represent values indexed to control. (G) Quantification of the western blot presented in (Fig 4G): Signal of the anti-p53 western blot is compared to anti-Actin signal. Data represent values indexed to control. Underlying data can be found in S1 Data. Cy3, anti-cleaved caspase-3 antibody; siRNA, small interfering RNA; TrkC-KF, TrkC killer-fragment.
(TIF)

S5 Fig TrkC-KF and Hey1 cooperate to inhibit MDM2 transcription by direct binding on its promoter. (A) Proximity ligation assay (DuoLink) using an anti-MDM2 antibody (recognizing endogenous MDM2) on SHEP cells transfected with TrkC-KF-Flag (anti-Flag antibody) or not transfected (anti-Hey1 antibody targeting endogenous Hey1 and anti-p53 antibody targeting endogenous p53): The protein–protein interactions are visualized by red fluorescent spots (Cy3). (B) Quantification of the western blot presented in Fig 5C, which has been reproduced and quantified 3 times: MDM2 signal is compared to actin signal. Data represent values indexed to control. (C) Quantification of cell death by trypan blue exclusion in SHEP cells transfected with siRNA control or siRNA Hey1 and plasmids encoding TrkC-FL and COBRA1. Data represent mean ± SEM (*n* = 3). **p < 0.01. *t* test. (D) Quantification of cell death by trypan blue exclusion in SHEP cells transfected with siRNA control or siRNA COBRA1 and plasmids encoding TrkC-FL and Hey1. Data represent mean ± SEM (*n* = 3). *t* test. (E) Human COBRA1 mRNA expression was assessed by RT-QPCR on SHEP cells transfected with siRNA control, siRNA NT-3, siRNA Hey1, or siRNA p53. Data represent mean ± SEM (*n* = 3) relative to GAPDH mRNA expression (housekeeping gene) and indexed to control. *p < 0.05. Two-sided Mann-Whitney test compared to control. (F,H,J) Schematic representation of *COBRA1* promoter (F), *BAX* promoter (H), and *BAK* promoter (J). Exons are indicated as gray boxes. The p53 binding site is indicated as a black box “P,” with the corresponding sequence. The couples of primers a, b, and c used in the ChIP experiments described in figures (G), (I), and (K) are also indicated. (G,I,K) ChIP assays using chromatin isolated from SHEP cells, which were untransfected (white bars), transfected with control (gray bars), or TrkC-KF and Hey1 constructs (black bars). Proteins were immunoprecipitated with an isotypic control antibody (white bars) or an anti-p53 antibody. DNA sequences from *COBRA1* promoter (G), *BAX* promoter, (I) and *BAK* promoter (K) were amplified by RT-QPCR using couples of primers a (upstream control), b (p53 binding site), and c (downstream control) indicated on (F,H,J). Data represent mean values ± SEM (*n* = 3) indexed to isotypic IgG value. Underlying data can be found in S1 Data. BAK, B cell lymphoma 2 killer; BAX, B cell lymphoma 2–associated X; ChIP, chromatin immunoprecipitation; COBRA1, cofactor of breast cancer 1; IgG, immunoglobulin G; MDM2, mouse double minute 2 homolog; ns, nonsignificant; NT-3, neurotrophin-3; siRNA, small interfering RNA; RT-QPCR, quantitative real-time PCR; TrkC, tropomyosin receptor kinase C; TrkC-FL, full-length TrkC; TrkC-KF, TrkC killer-fragment.
(TIF)

S6 Fig NB tumors gain a selective advantage when the TrkC apoptotic pathway is silenced. (A, B) TrkC and Hey1 expression in neuroblastic tumors. Dot plots of TrkC (A) and Hey1 (B) mRNA expression values in neuroblastic tumor samples stages 1 to 3 versus stage 4 analyzed using the Agilent microarray 44K (T. Wolf cohort [54], 649 samples, NB1–3 [*n* = 357], and NB4 [*n* = 214]). For (A, B) statistical analysis, a 2-sided Mann-Whitney nonparametric test was used to compare the expression values corresponding to NB1–3 versus NB4. * *p* < 0.05. **** *p* < 0.0001. (C) Analysis of expression data obtained with Agilent-Microarray 44K on T. Wolf cohort. Tumors in which expression of the corresponding gene is below the intergrade median are considered as "low." Tumors in which expression of the corresponding gene is above the intergrade median are considered as "high." Tumors with a NT-3^low^, TrkC^high^, and Hey^high^ or a NT-3^low^, TrkC^low^, and Hey^low^ profile were counted and indicated as a percentage. "Others" encompasses tumors (NT-3^high^, TrkC^high^, Hey^low^), (NT-3^high^, TrkC^high^, Hey^high^), (NT-3^high^, TrkC^low^, Hey^low^), or (NT-3^high^, TrkC^low^, Hey^high^). (D) Kaplan-Meier survival curve based on T. Wolf cohort. Tumors were classified as in (C) to form groups A (NT-3^low^, TrkC^high^, Hey1^high^; *n* = 81), B (NT-3^low^, TrkC^low^, Hey1^low^; *n* = 47), and C (others; *n* = 287). For 234 samples of the cohort, patients’ survival information was not available. Group A (NT-3^low^, TrkC^high^, Hey1^high^; *n* = 81) shows a better event-free survival compared to Group B (NT-3^low^, TrkC^low^, Hey1^low^; *n* = 47) and Group C (others; *n* = 287), in which TrkC apoptotic signaling is impaired. Logrank *p* = 0.059. Underlying data can be found in S1 Data. NB, neuroblastoma; NT-3, neurotrophin-3; TrkC, tropomyosin receptor kinase C.
(TIF)

S7 Fig Schematic representation of TrkC proapoptotic pathway. When TrkC is deprived of its ligand, its intracellular domain is double cleaved by caspase, and the released fragment is called TrkC-KF. TrkC-KF is shuttled into the nucleus by importins and interacts there with Hey1 bHLH transcription factor. Hey1 and TrkC-KF bind jointly on *MDM2* promoter and deregulate MDM2 expression, consequently stabilizing p53. Among other putative functions, p53 transactivates the expression of COBRA1 and BAX. COBRA1 shuttles TrkC-KF to the mitochondria where it activates BAX, induces MOMP, cytochrome *c* release, and the subsequent apoptosome activation. BAX, B cell lymphoma 2–associated X; bHLH, basic helix-loop-helix; COBRA1, cofactor of breast cancer 1; MDM2, mouse double minute 2 homolog; MOMP, mitochondrial outer membrane permeabilization; TrkC, tropomyosin receptor kinase C; TrcK-KF, TrkC killer-fragment.
(TIF)

## Abbreviations

ALK^F1178L^: anaplastic lymphoma kinase F1178L
BAK: B cell lymphoma 2 killer
BAX: B cell lymphoma 2–associated X
BDNF: brain-derived neurotrophic factor
bHLH: basic helix-loop-helix
bHLH-O: bHLH-Orange
CAM: chorioallantoic membrane
CAS9: CRISPR-associated protein 9
ChIP: chromatin immunoprecipitation
ChIP-Seq: ChIP sequence
COBRA1: cofactor of breast cancer 1
cPARP: poly [ADP-ribose] polymerase cleavage
CRISPR: clustered regularly interspaced short palindromic repeat
DBD: DNA-binding domain
DCC-IC: deleted in colorectal cancer intracellular domain
E-box: enhancer box
GEO: Gene Expression Omnibus
GFP: green fluorescent protein
gRNA: guide RNA
KPNA4: karyopherin alpha 4
MAPK: mitogen-activated protein kinase
MDM2: mouse double minute 2 homolog
MOMP: mitochondrial outer membrane permeabilization
N2A: Neuro2a
NB: neuroblastoma
NCBI: the National Center for Biotechnology Information
NCC: neural crest cell
Neo-IC: intracellular fragment of Neogenin
Neo-IC-ΔNES: Neo-IC deleted for its nuclear export sequence
NES: nuclear export sequence
NGF: nerve growth factor
NLS: nuclear localization sequence
ns: nonsignificant
NT-3: neurotrophin-3
NTRK3: neurotrophin-3 receptor tyrosine kinase receptor C
p75^NTR^: neurotrophin receptor p75
PARP: poly [ADP-ribose] polymerase
PI3K: phosphoinositide 3-kinase
Ptc: Patched
RFP: red fluorescent protein
RTK: tyrosine kinase receptor
RT-QPCR: quantitative real-time PCR
siRNA: small interfering RNA
TrkA: tropomyosin receptor kinase A
TrkC: tropomyosin receptor kinase C
TrkC-FL: full-length TrkC
TrkC-IC-DM: intracellular domain of TrkC mutated on D495N and D641N
TrkC-KF: TrkC killer-fragment
TrkC-KF-GFP: TrkC-KF tagged with GFP
UAS: upstream activating sequence
