# Supplementary material for: Hey1- and p53-dependent TrkC proapoptotic activity controls neuroblastoma growth
Source: PLoS Biol. 2018 May 11;16(5):e2002912. doi: 10.1371/journal.pbio.2002912 (PMC5965893; doi:10.1371/journal.pbio.2002912)
Supplement: S1 Table — RT-QPCR was performed using the TaqMan technique, requiring the indicated probes (Universal Probe Library, Roche Applied Science). RT-QPCR, quantitative real-time PCR. (XLS) [file pbio.2002912.s002.xls]

**Supplementary Table 1**

| **Gene** | **sens** | **Primer 5'-3'** | **probe** |
| --- | --- | --- | --- |
| human TrkC | forward | atgcttgtggctgtgaagg | 71 |
|  | reverse | catgctgcaggttggtga |  |
| human NT-3 | forward | aaaaacggttgcaggggtat | 47 |
|  | reverse | ggtttgggatgttttgcact |  |
| human Cobra1 | forward | tctcgatggcgtcaagaag | 68 |
|  | reverse | cacacaggatcatggacaggt |  |
| human Hey1 | forward | catacggcaggagggaaag | 29 |
|  | reverse | gcatctagtccttcaatgatgct |  |
| human MDM2 | forward | ccatgatctacaggaacttggtagta | 18 |
|  | reverse | tcactcacagatgtacctgagtcc |  |
| Gal4 | forward | tgaataaagatgccgtcacaga | 139 |
|  | reverse | tgtctcaatgttagaggcatatcag |  |
| human DCC-IC | forward | tccaaagttgccaagacctc | 5 |
|  | reverse | ttcccagttgggtttctgac |  |
| human TrkC-KF | forward | caattgccacaagccagac | 91 |
|  | reverse | cccagttctctcttcaacacg |  |
| human TrkC-495-825 | forward | gaaggccaccccaatctac | 142 |
|  | reverse | tataaacgcttggccacca |  |
| rat Hey1 | forward | catgaagagagctcacccaga | 17 |
|  | reverse | gaacacagagccgaactcaa |  |
| rat Hey2 | forward | gtggggagcgagaacaatta | 104 |
|  | reverse | gttgtcggtgaattggacct |  |
| rat HeyL | forward | ggctgtcaggctggataaaa | 25 |
|  | reverse | gaagagccgcctctgatg |  |
| human Neogenin | forward | acaccagatgttgctgttcg | 48 |
|  | reverse | tccaaggacagattctgagga |  |
| human HPRT | forward | tgaccttgatttattttgcatacc | 73 |
|  | reverse | cgagcaagacgttcagtcct |  |
| mouse HPRT | forward | tcctcctcagaccgctttt | 95 |
|  | reverse | cctggttcatcatcgctaatc |  |
| human GAPDH | forward | ccccggtttctataaattgagc | 63 |
|  | reverse | caccttccccatggtgtct |  |
