# Supplementary material for: Hey1- and p53-dependent TrkC proapoptotic activity controls neuroblastoma growth
Source: PLoS Biol. 2018 May 11;16(5):e2002912. doi: 10.1371/journal.pbio.2002912 (PMC5965893; doi:10.1371/journal.pbio.2002912)
Supplement: S2 Table — RT-QPCR was performed using the TaqMan technique, requiring the indicated probes (Universal Probe Library, Roche Applied Science). ChIP, chromatin immunoprecipitation; RT-QPCR, quantitative real-time PCR. (XLS) [file pbio.2002912.s003.xls]

**Supplementary Table 2.**

| **promoter** | **primers** | **sens** | **sequence 5' - 3'** | **probe** |
| --- | --- | --- | --- | --- |
| MDM2 promoter | a | forward | ggaagtttcctttctggtaggc | 7 |
|  |  | reverse | ccttcccttctgcagcttt |  |
|  | b | forward | gactccaagcgcgaaaac | 68 |
|  |  | reverse | gcccagacccaaaagtgac |  |
|  | c | forward | cctggttagtatttttgtctcgtgt | 21 |
|  |  | reverse | gcacactttaagctatgcacataca |  |
| Cobra1 promoter | a | forward | gccctgcagactccactc | 3 |
|  |  | reverse | caggccagtgatgacaagg |  |
|  | b | forward | tgctggagcgagtgtcag | 30 |
|  |  | reverse | agtcgatccaaggctgagag |  |
|  | c | forward | tttgtagaggtggggtctgg | 26 |
|  |  | reverse | aaggctgatgtgggaggat |  |
| Bax promoter | a | forward | gcgaggatgcttgagtctg | 54 |
|  |  | reverse | ggagatgaggtctctctatgtgc |  |
|  | b | forward | tgggctcacaagttaggacaa | 85 |
|  |  | reverse | ccaggcaggacgttatagatg |  |
|  | c | forward | gctccttcaggacacaggac | 63 |
|  |  | reverse | ggggcccagactcctagtt |  |
| Bak promoter | a | forward | ggaagggtcttgtccatcct | 23 |
|  |  | reverse | ccttgagcttccccttcc |  |
|  | b | forward | ctgcccaacatccctagact | 61 |
|  |  | reverse | gacttcctgggcttgctg |  |
|  | c | forward | Cggctgcaaagttctgtttt | 25 |
|  |  | reverse | ggacagggtgaaggcagac |  |
