# Supplementary figures and images for: Hey1- and p53-dependent TrkC proapoptotic activity controls neuroblastoma growth
Source: PLoS Biol. 2018 May 11;16(5):e2002912. doi: 10.1371/journal.pbio.2002912 (PMC5965893; doi:10.1371/journal.pbio.2002912)

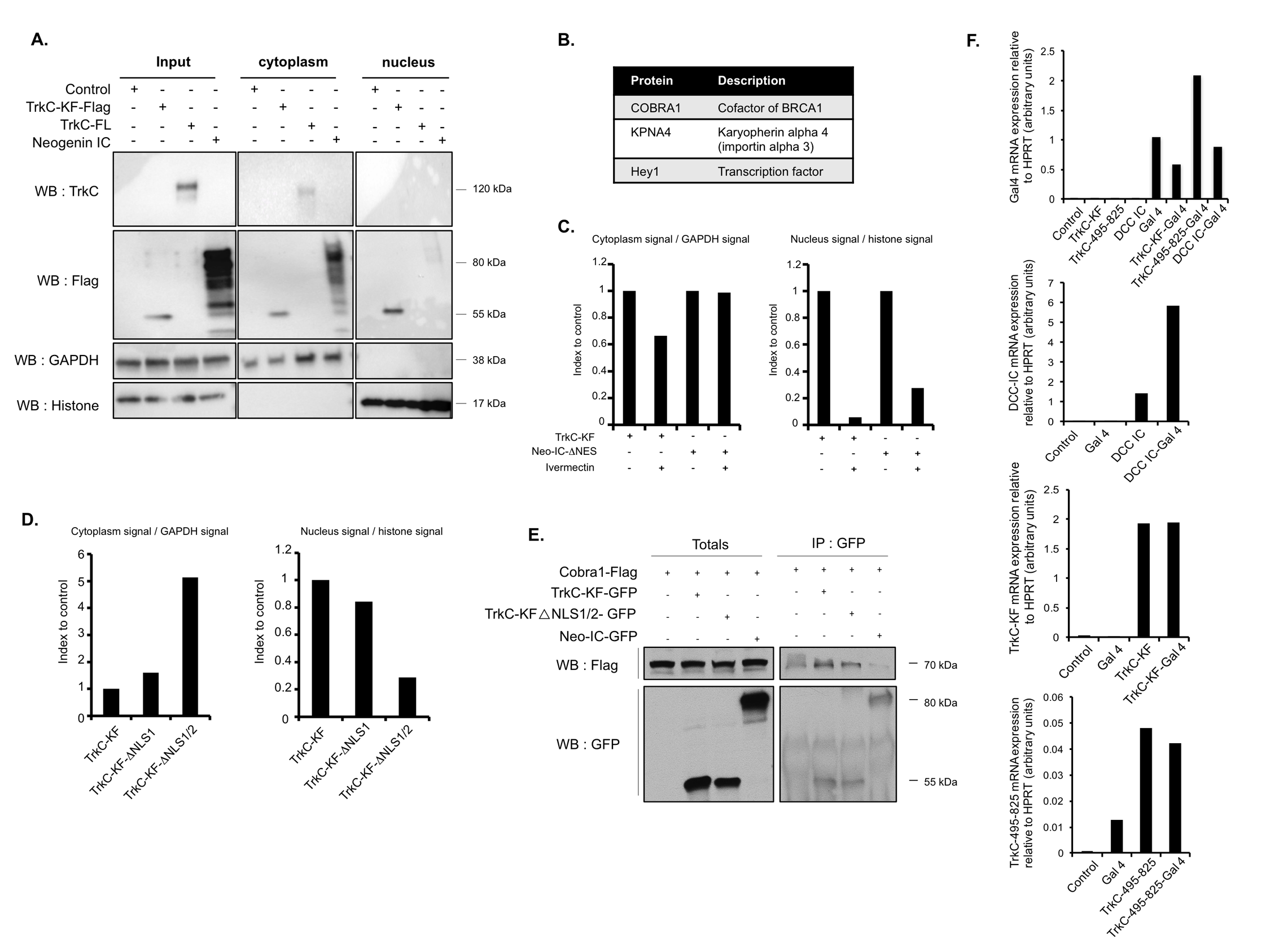

Supplement: S1 Fig — (A) SHEP cells transfected with either control plasmid, TrkC-KF-Flag, TrkC-FL, or Neo-IC were fractionated into cytoplasmic (Cytoplasm, marker: GAPDH) and nuclear (Nucleus, marker: histone) fractions. Input corresponds to the construct expression in whole cell lysates. (B) Candidate partners for TrkC-KF obtained in the 2-hybrid screen assay. (C) Quantification of the western blots presented in (Fig 1E): Signal of the anti-GFP western blot is compared to GAPDH signal (for the input and cytoplasmic fraction) and Histone H3 signal (for the nuclear fraction). Data represent values indexed to control (TrkC-KF). (D) Quantification of the western blots presented in (Fig 1G): Signal of the anti-GFP western blot is compared to GAPDH signal (for the input and cytoplasmic fraction) and Histone H3 signal (for the nuclear fraction). Data represent values indexed to control (TrkC-KF). (E) IP of TrkC-KF-GFP and TrkC-KF-ΔNLS1/2-GFP using an anti-GFP antibody in HEK293T-transfected cells. COBRA1 is tagged with a Flag epitope. Neo-IC-GFP is used as unrelated negative control. (F) Gal4, DCC-IC, TrkC-KF, and TrkC-495-825 mRNA expression were assessed by RT-QPCR to verify the expression of constructs used in the luciferase assay presented in Fig 1I. Data represent values (arbitrary units) relative to HPRT mRNA expression (housekeeping gene). Underlying data can be found in S1 Data. COBRA1, cofactor of breast cancer 1; DCC-IC, deleted in colorectal cancer intracellular domain; GAPDH, glyceraldehyde 3-phosphate dehydrogenase; GFP, green fluorescent protein; HEK293T, human embryonic kidney 293 T; HPRT, hypoxanthine phosphoribosyltransferase; IP, immunoprecipitation; KPNA4, karyopherin alpha 4; Neo-IC-GFP, Neogenin intracellular domain tagged with GFP; NLS, nuclear localization sequence; RT-QPCR, quantitative real-time PCR; TrkC, tropomyosin receptor kinase C; TrkC-FL, full-length TrkC; TrkC-KF, TrkC killer-fragment. (TIF) [file pbio.2002912.s004.tif]

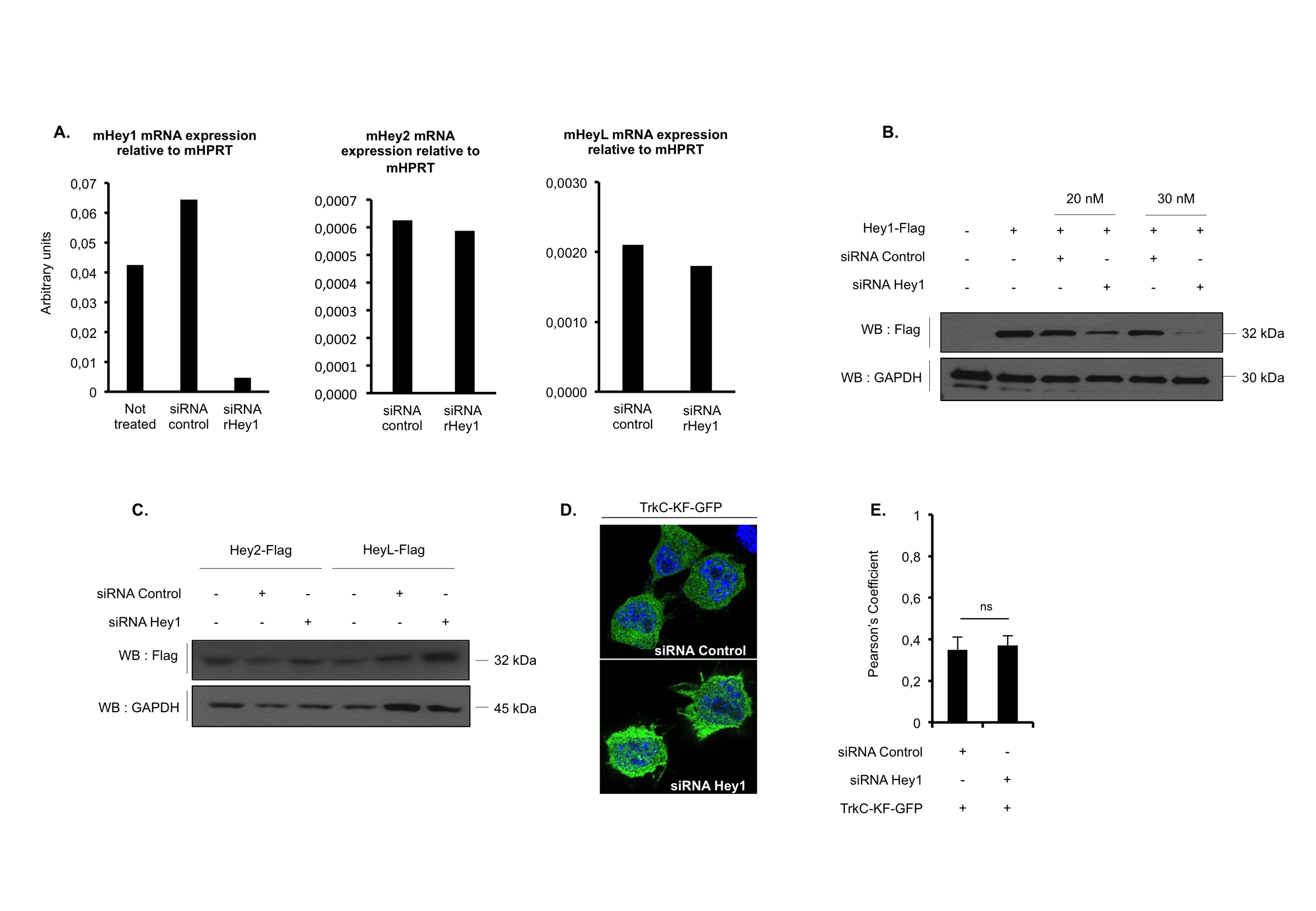

Supplement: S2 Fig — (A) Mouse Hey1, Hey2, and HeyL mRNA expression were assessed in N2A cells transfected with an siRNA control or an siRNA targeting Hey1. Data represent values (arbitrary units) relative to HPRT mRNA expression (housekeeping gene). (B) Hey1 expression was assessed by western blot in N2A cells transfected with a Hey1-Flag expression construct and an siRNA control or an siRNA Hey1 at 2 different concentrations (20 nM and 30 nM). GAPDH is used as a loading control. (C) Hey2 and HeyL expression was assessed by western blot in N2A cells transfected with Hey2-Flag and HeyL-Flag constructs and an siRNA control or an siRNA targeting Hey1 at 30 nM. Actin is used as a loading control. (D,E) Expression of TrkC-KF-GFP in N2A cells, transfected with an siRNA control or an siRNA targeting Hey1, indicates a partial localization in the nucleus, as shown by confocal analysis (A) and by the associated Pearson’s coefficient (B), in presence or absence of Hey1. Data represent mean ± SEM (3 independent fields). t test compared to control (TrkC-GFP + siRNA control). Underlying data can be found in S1 Data. GAPDH, glyceraldehyde 3-phosphate dehydrogenase; GFP, green fluorescent protein; HPRT, hypoxanthine phosphoribosyltransferase; N2A, Neuro2a; ns, nonsignificant; siRNA, small interfering RNA; TrkC, tropomyosin receptor kinase C; TrkC-KF, TrkC killer-fragment. (TIF) [file pbio.2002912.s005.tif]

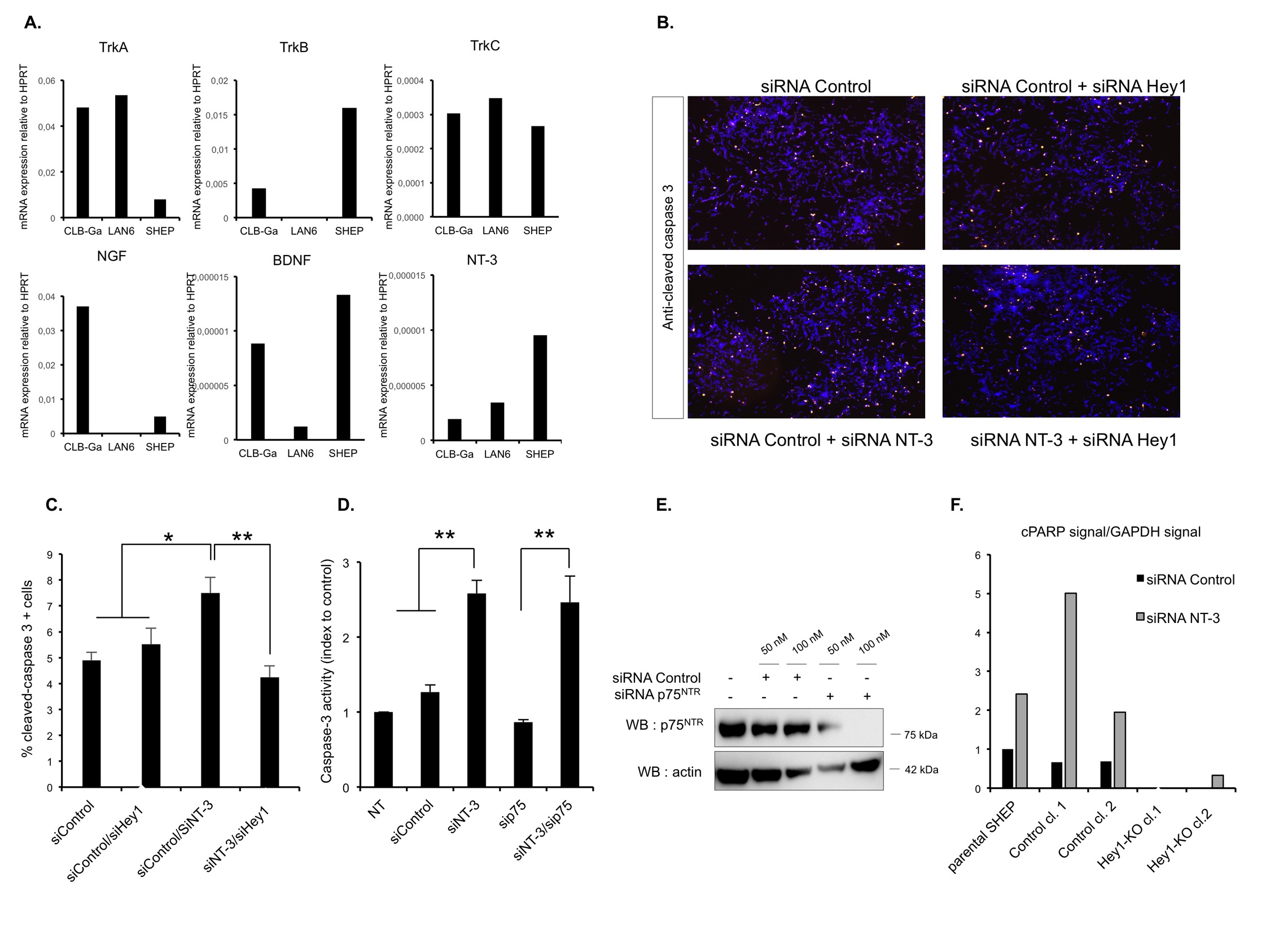

Supplement: S3 Fig — (A) TrkA, TrkB, TrkC, NGF, BDNF, and NT-3 mRNA expression was assessed by RT-QPCR on CLB-Ga, LAN6, and SHEP cells relative to HPRT mRNA expression (housekeeping gene). A representative experiment is shown. (B) Immunofluorescence staining using Cy3 performed on LAN6 cells transfected or with the indicated siRNA. A representative picture is shown for each condition. Nuclei are stained with DAPI. (C) Quantification of the Cy3 staining shown in (B) as a percentage of total cell number measured by DAPI staining. Data represent mean ± SEM (n = 3 independent fields). (D) Caspase-3 activity assay on SHEP cells transfected with siRNA control, siRNA NT-3, and siRNA p75NTR (p75). Data represent mean ± SEM (n = 3) indexed to control. **p < 0.01. t test. (E) p75NTR expression was assessed by western blot in N2A cells transfected with a p75NTR expression construct and an siRNA control or an siRNA p75NTR at 2 different concentrations (50 nM and 100 nM). Actin is used as a loading control. (F) Quantification of the western blot presented in (Fig 3F): signal of the anticleaved PARP western blot is compared to anti-GAPDH signal. Data represent values indexed to control (parental SHEP transfected with siRNA control). Underlying data can be found in S1 Data. BDNF, brain-derived neurotrophic factor; Cy3, anticleaved caspase-3 antibody; GAPDH, glyceraldehyde 3-phosphate dehydrogenase; HPRT, hypoxanthine phosphoribosyltransferase; NGF, nerve growth factor; N2A, Neuro2a; NT-3, neurotrophin-3; p75NTR; neurotrophin receptor p75; PARP, poly [ADP-ribose] polymerase; RT-QPCR, quantitative real-time PCR; siRNA, small interfering RNA; TrkC, tropomyosin receptor kinase C. (TIF) [file pbio.2002912.s006.tif]

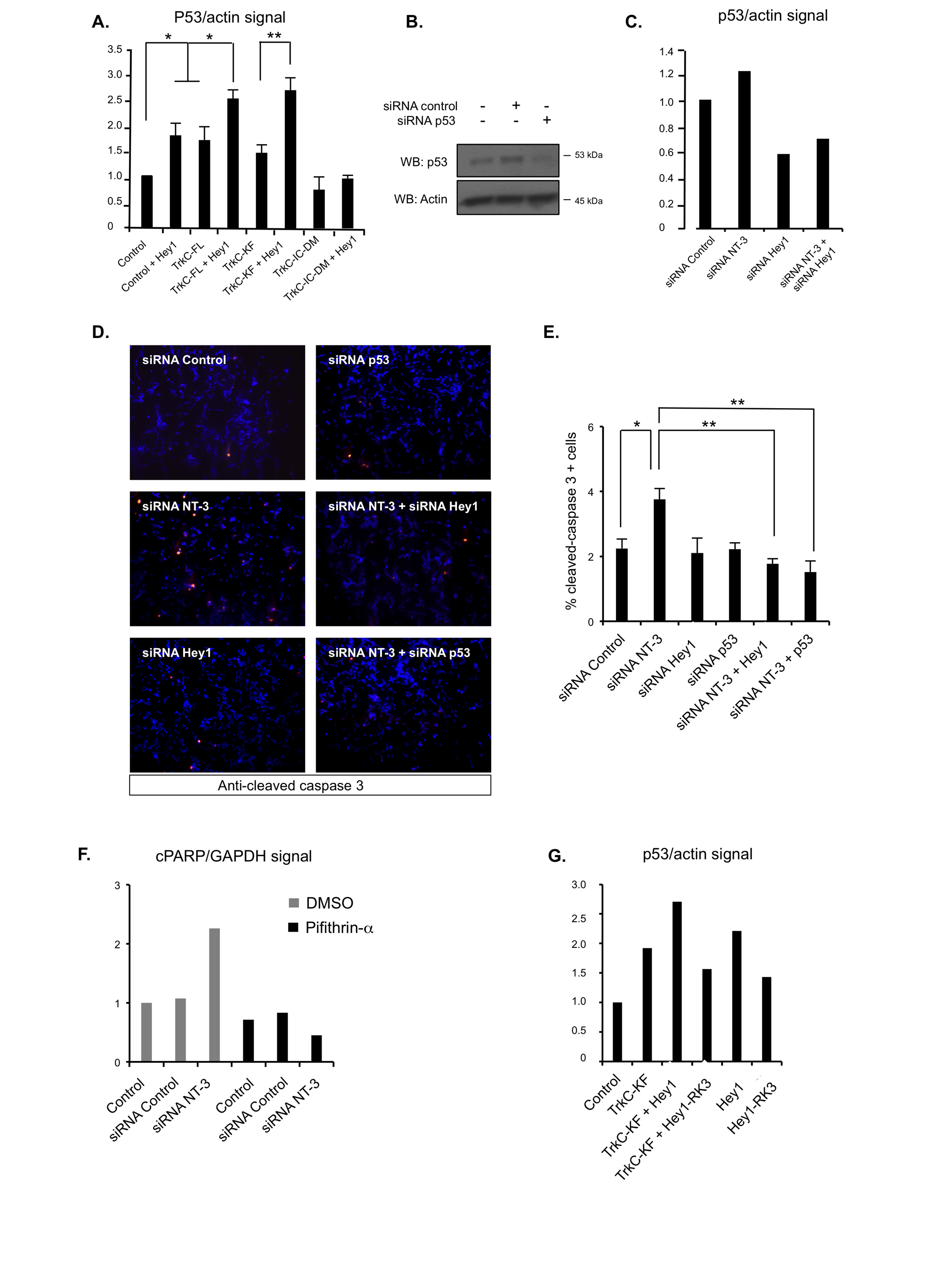

Supplement: S4 Fig — (A) Quantification of the western blot presented in (Fig 4A), which has been reproduced and quantified 3 times: Signal of the anti-p53 western blot is compared to anti-Actin signal. Data represent values indexed to control, mean ± SEM (n = 3). *p < 0.05, **p < 0.01. t test. (B) p53 expression was assessed by western blot in SHEP cells transfected with an siRNA control and an siRNA p53 at 100 nM. Actin is used as a loading control. (C) Quantification of the western blots presented in (Fig 4D): Signal of the anti-p53 western blot is compared to anti-Actin signal. Data represent values indexed to siRNA control. (D) Immunofluorescence staining using Cy3 performed on CLB-Ga cells transfected or with the indicated siRNA. A representative picture is shown for each condition. Nuclei are stained with DAPI. (E) Quantification of Cy3 staining shown in (D) as a percentage of total cell number measured by DAPI staining. Data represent mean ± SEM (n = 3 independent fields). *p < 0.05, **p < 0.01. t test. (F) Quantification of the western blot presented in (Fig 4F): Signal of the anti-p53 western blot is compared to anti-Actin signal. Data represent values indexed to control. (G) Quantification of the western blot presented in (Fig 4G): Signal of the anti-p53 western blot is compared to anti-Actin signal. Data represent values indexed to control. Underlying data can be found in S1 Data. Cy3, anti-cleaved caspase-3 antibody; siRNA, small interfering RNA; TrkC-KF, TrkC killer-fragment. (TIF) [file pbio.2002912.s007.tif]

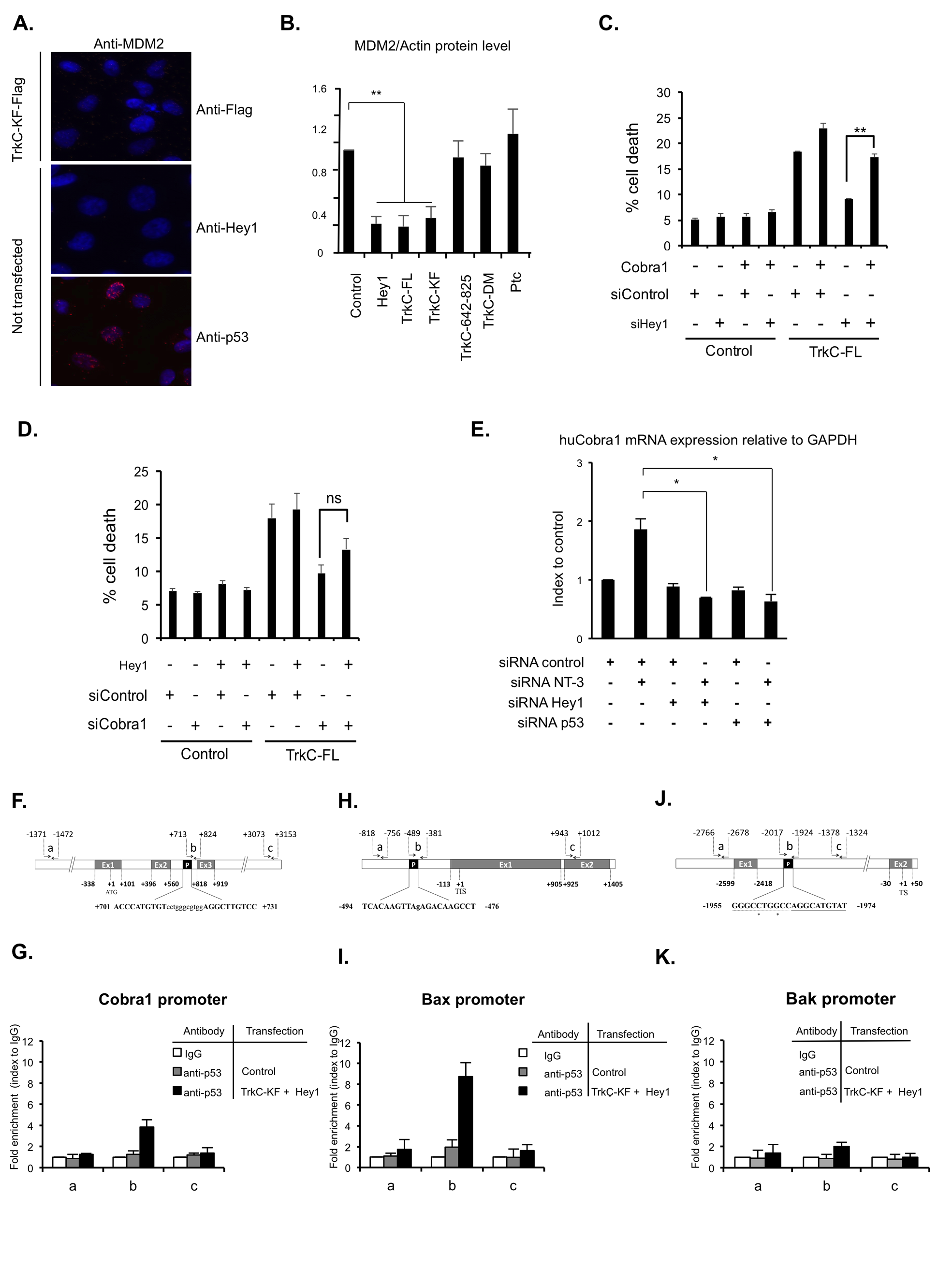

Supplement: S5 Fig — (A) Proximity ligation assay (DuoLink) using an anti-MDM2 antibody (recognizing endogenous MDM2) on SHEP cells transfected with TrkC-KF-Flag (anti-Flag antibody) or not transfected (anti-Hey1 antibody targeting endogenous Hey1 and anti-p53 antibody targeting endogenous p53): The protein–protein interactions are visualized by red fluorescent spots (Cy3). (B) Quantification of the western blot presented in Fig 5C, which has been reproduced and quantified 3 times: MDM2 signal is compared to actin signal. Data represent values indexed to control. (C) Quantification of cell death by trypan blue exclusion in SHEP cells transfected with siRNA control or siRNA Hey1 and plasmids encoding TrkC-FL and COBRA1. Data represent mean ± SEM (n = 3). **p < 0.01. t test. (D) Quantification of cell death by trypan blue exclusion in SHEP cells transfected with siRNA control or siRNA COBRA1 and plasmids encoding TrkC-FL and Hey1. Data represent mean ± SEM (n = 3). t test. (E) Human COBRA1 mRNA expression was assessed by RT-QPCR on SHEP cells transfected with siRNA control, siRNA NT-3, siRNA Hey1, or siRNA p53. Data represent mean ± SEM (n = 3) relative to GAPDH mRNA expression (housekeeping gene) and indexed to control. *p < 0.05. Two-sided Mann-Whitney test compared to control. (F,H,J) Schematic representation of COBRA1 promoter (F), BAX promoter (H), and BAK promoter (J). Exons are indicated as gray boxes. The p53 binding site is indicated as a black box “P,” with the corresponding sequence. The couples of primers a, b, and c used in the ChIP experiments described in figures (G), (I), and (K) are also indicated. (G,I,K) ChIP assays using chromatin isolated from SHEP cells, which were untransfected (white bars), transfected with control (gray bars), or TrkC-KF and Hey1 constructs (black bars). Proteins were immunoprecipitated with an isotypic control antibody (white bars) or an anti-p53 antibody. DNA sequences from COBRA1 promoter (G), BAX promoter, (I) and BAK promoter (K) were amplifi [file pbio.2002912.s008.tif]

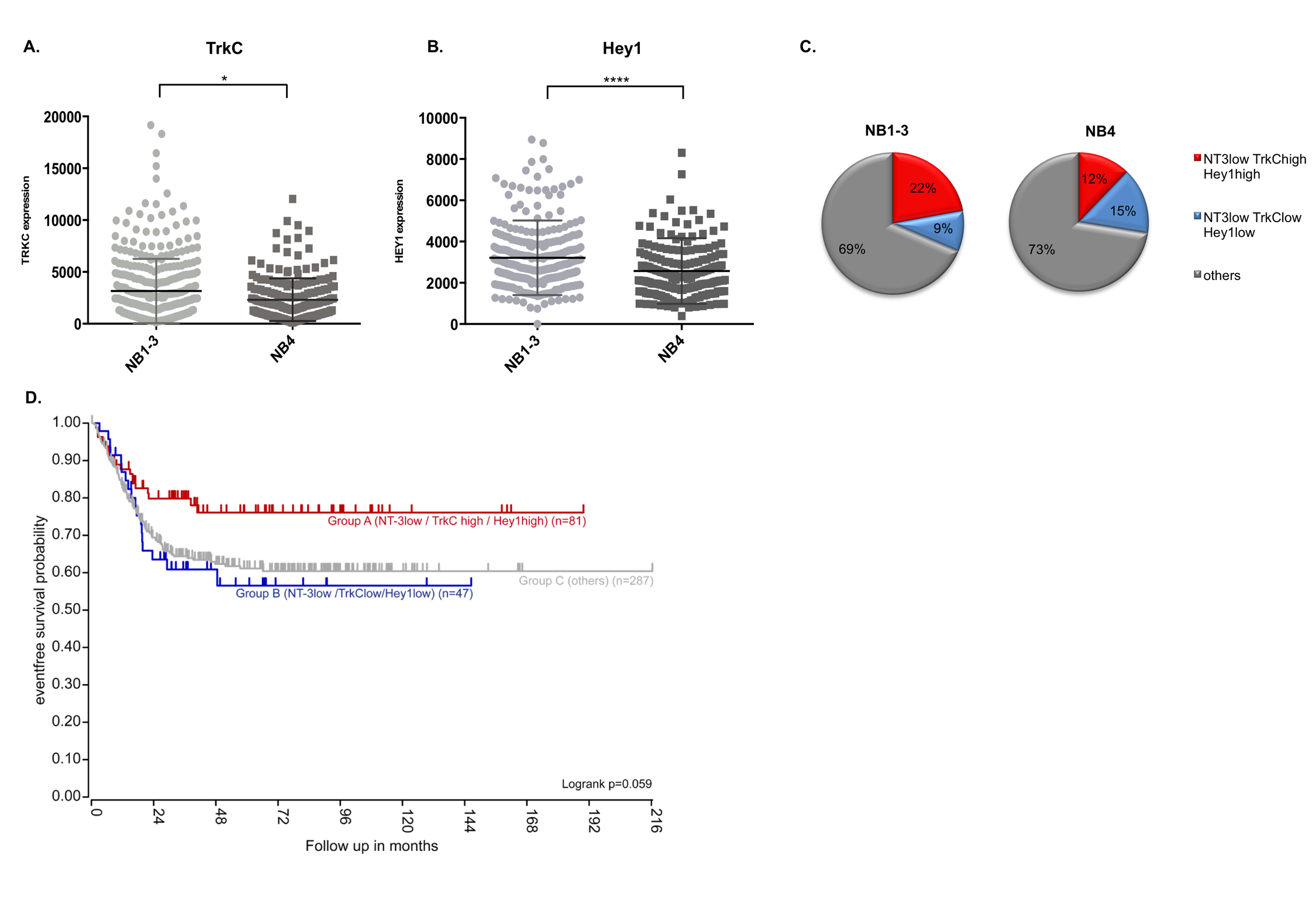

Supplement: S6 Fig — (A, B) TrkC and Hey1 expression in neuroblastic tumors. Dot plots of TrkC (A) and Hey1 (B) mRNA expression values in neuroblastic tumor samples stages 1 to 3 versus stage 4 analyzed using the Agilent microarray 44K (T. Wolf cohort [54], 649 samples, NB1–3 [n = 357], and NB4 [n = 214]). For (A, B) statistical analysis, a 2-sided Mann-Whitney nonparametric test was used to compare the expression values corresponding to NB1–3 versus NB4. * p < 0.05. **** p < 0.0001. (C) Analysis of expression data obtained with Agilent-Microarray 44K on T. Wolf cohort. Tumors in which expression of the corresponding gene is below the intergrade median are considered as "low." Tumors in which expression of the corresponding gene is above the intergrade median are considered as "high." Tumors with a NT-3low, TrkChigh, and Heyhigh or a NT-3low, TrkClow, and Heylow profile were counted and indicated as a percentage. "Others" encompasses tumors (NT-3high, TrkChigh, Heylow), (NT-3high, TrkChigh, Heyhigh), (NT-3high, TrkClow, Heylow), or (NT-3high, TrkClow, Heyhigh). (D) Kaplan-Meier survival curve based on T. Wolf cohort. Tumors were classified as in (C) to form groups A (NT-3low, TrkChigh, Hey1high; n = 81), B (NT-3low, TrkClow, Hey1low; n = 47), and C (others; n = 287). For 234 samples of the cohort, patients’ survival information was not available. Group A (NT-3low, TrkChigh, Hey1high; n = 81) shows a better event-free survival compared to Group B (NT-3low, TrkClow, Hey1low; n = 47) and Group C (others; n = 287), in which TrkC apoptotic signaling is impaired. Logrank p = 0.059. Underlying data can be found in S1 Data. NB, neuroblastoma; NT-3, neurotrophin-3; TrkC, tropomyosin receptor kinase C. (TIF) [file pbio.2002912.s009.tif]

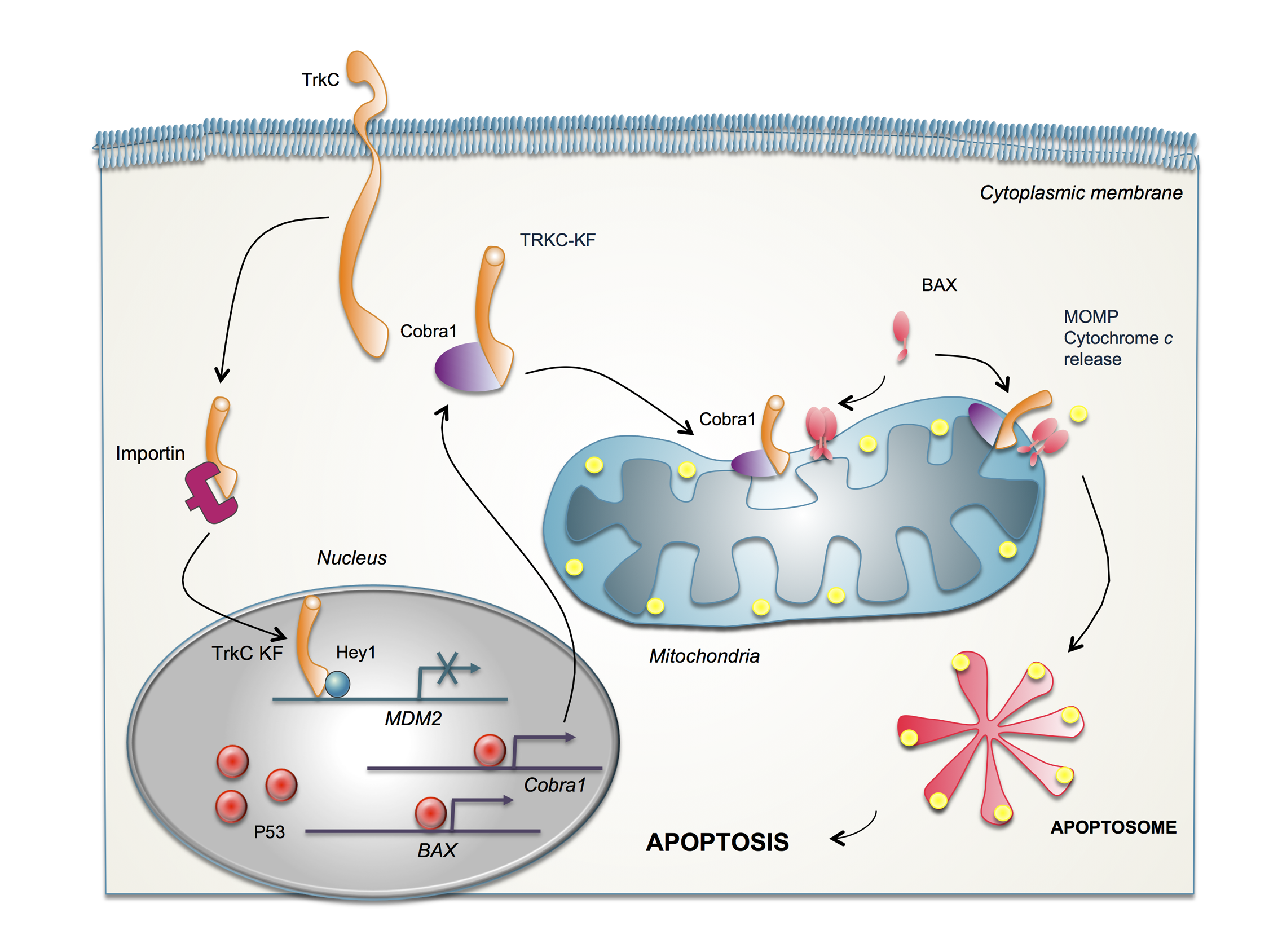

Supplement: S7 Fig — When TrkC is deprived of its ligand, its intracellular domain is double cleaved by caspase, and the released fragment is called TrkC-KF. TrkC-KF is shuttled into the nucleus by importins and interacts there with Hey1 bHLH transcription factor. Hey1 and TrkC-KF bind jointly on MDM2 promoter and deregulate MDM2 expression, consequently stabilizing p53. Among other putative functions, p53 transactivates the expression of COBRA1 and BAX. COBRA1 shuttles TrkC-KF to the mitochondria where it activates BAX, induces MOMP, cytochrome c release, and the subsequent apoptosome activation. BAX, B cell lymphoma 2–associated X; bHLH, basic helix-loop-helix; COBRA1, cofactor of breast cancer 1; MDM2, mouse double minute 2 homolog; MOMP, mitochondrial outer membrane permeabilization; TrkC, tropomyosin receptor kinase C; TrcK-KF, TrkC killer-fragment. (TIF) [file pbio.2002912.s010.tif]
